# PTEN variant and genetic backgrounds combine to modify cerebellar neuronal differentiation in autism spectrum disorder

**DOI:** 10.1093/hmg/ddaf185

**Published:** 2025-12-10

**Authors:** Ya Chen, Shuai Fu, Timothy P H Sit, Anthony Wynshaw-Boris

**Affiliations:** Department of Genetics and Genome Sciences, CWRU School of Medicine, 10900 Euclid Ave., BRB721, Cleveland, OH 44106, United States; Department of Genetics and Genome Sciences, CWRU School of Medicine, 10900 Euclid Ave., BRB721, Cleveland, OH 44106, United States; Genomic Analysis Laboratory, The Salk Institute for Biological Studies, 10010 North Torrey Pines Road, La Jolla, CA 92037, United States; Department of Physiology, Development and Neuroscience, University of Cambridge, 7 Downing Place, Cambridge, CB2 3DY, United Kingdom; Sainsbury Wellcome Centre, University College London, 25 Howland St. London, W1T 4JG, United Kingdom; Department of Genetics and Genome Sciences, CWRU School of Medicine, 10900 Euclid Ave., BRB721, Cleveland, OH 44106, United States

**Keywords:** Cerebellar organoids, Autism spectrum disorder, PTEN variant, Single cell RNA sequencing

## Abstract

Mutations in the *PTEN* gene have been implicated in autism spectrum disorders (ASD), particularly among individuals with comorbid macrocephaly. In our previous study, we demonstrated that the *PTEN* p.Ile135Leu variant, in an ASD-related genetic background dependent fashion, disrupts both cortical neurogenesis and gliogenesis. While abnormal cerebellar development is a recognized feature of ASD, the specific cellular targets and timing of disruptions during cerebellar differentiation and development remain poorly understood. To investigate these aspects, we applied our previously established cerebellar organoid protocol and used isogenic human iPSC lines harboring this *PTEN*-variant. We examined the expression of Purkinje cells, granule cells, interneurons, and glial cells prior to 22 weeks of differentiation, assessed genes expression at 8 weeks, and evaluated spontaneous spikes activity in Purkinje cells after 11 weeks. We observed that cell-type-specific expression patterns differed between the *PTEN* p.Ile135Leu variant in control versus ASD-genetic backgrounds. However, these background differences were diminished in *PTEN* knockout lines across both backgrounds. Our single-cell RNA sequencing (scRNA-seq) dataset revealed that the *PTEN* p.Ile135Leu variant increased the number of interneuron progenitor cells, whereas *PTEN* knockout led to an expansion of meningeal-like cells in both genetic contexts. Moreover, both the *PTEN* p.Ile135Leu variant and *PTEN* knockout abolished spontaneous simple spikes activity in Purkinje cells across both backgrounds, including *PTEN*-corrected patient-derived lines. Together, these findings provide direct evidence linking *PTEN* dysfunction and genetic background to altered cerebellar differentiation and neuronal network activity in human cerebellar organoids.

## Introduction

The phosphatase and tensin homologue (*PTEN*) gene functions as a tumor suppressor through its action on both lipid second messenger phosphatase and protein phosphatase activities [1, 2]. These functions inhibit cell migration and proliferation in the cytoplasm, while also regulating chromosome stability and DNA repair in the nucleus [3–5]. *PTEN* acts as a critical negative regulator of the PI3K/AKT/mTOR signaling pathway. Loss of the *PTEN* function through mutation or deletion leads to hyperactivation of this pathway, promoting increased cell proliferation, enhanced cell survival, and resistance to apoptosis [6].

Commensurate with these important functions, *PTEN* also plays an essential role in maintaining neural stem and progenitor cell populations. Consequently, *PTEN* is an important genetic cause of autism spectrum disorders (ASD), discovered when germline *PTEN* mutations were found in individuals with ASD and macrocephaly [7]. A subset of children with ASD exhibits a neurodevelopmental phenotype characterized by abnormal brain overgrowth during early life (6–24 months), often associated with disrupted neural development [8]. In the prenatal stages of mice, *PTEN* deficiency results in the retention of neural precursors that preferentially differentiate into neurons [9]. Postnatally, these same precursors display enhanced glial differentiation [10]. Moreover, *PTEN* disruption accelerates Bergmann glial differentiation and leads to increased astrocyte proliferation and morphological abnormalities [11].

Mouse models with *PTEN* mutations have been widely used to study ASD, providing valuable insights into how *PTEN* dysfunction contributed to early brain overgrowth, neuronal abnormalities, and behavioral phenotypes characteristic of ASD [12, 13]. Using haploinsufficient *PTEN* +/− mice, cortical cultures display increased proliferation of the glial cell population [14]. Modest cerebellar hypertrophy has been observed in heterozygous *PTEN* conditional knockout mice, accompanied by a higher glia-to-neuron ratio [15]. In the *PTEN*^m3m4/m3m4^ cytoplasmic-predominant mutant model, dysregulated *PTEN* function leads to increased microglia activation, neuronal soma hypertrophy, gliosis, and autistic-like behavior [16]. Several conditional *PTEN* knockout models, including *NESTIN*-Cre, *NKX2.1*-Cre, L7/*PCP2*-Cre, and *GFAP*-Cre mediated inactivation, have demonstrated that *PTEN* loss results in abnormalities in cell size, neurite outgrowth and cell degeneration, accompanied by macrocephaly, social deficits, and disrupted neuronal differentiation and development [15, 17–20]. Recent studies have documented cerebellar dysfunction and Purkinje cells deficit in ASD, with individuals carrying PTEN-ASD variants exhibiting not only cognitive and social impairments but also motor coordination deficits. Several studies have further investigated the role of PTEN in cerebellar development, highlighting its critical function in Purkinje cell development [21–24]. However, most of these studies were performed in animal models, as accessing human infancy for direct study remains challenging.

It has been difficult to study the role of *PTEN* in human brain development, which occurs during the first trimester of fetal development, and is inaccessible to detailed molecular study. Human induced pluripotent stem cells (iPSCs) models of *PTEN* mutations offer a powerful platform to study human cells during gestation and to bridge the gap between fetal clinical phenotypes and molecular mechanism in ASD. We have used these models to enable patient-specific studies that account for genetic background, support differentiation into relevant brain regions and cell types, and provide a valuable system for mechanistic investigation and drug screening [25, 26]. To more accurately model human ASD, brain organoids offer a three-dimensional environment that better recapitulates the structural and cellular complexity of the human brain compared to traditional two-dimensional cultures [27–32]. Most published 3D *PTEN* organoids studies have been limited to cerebral organoids, with significantly fewer studies focusing on *PTEN*-mutant cerebellar organoids, despite the cerebellum’s known involvement in ASD.

The cerebellum, traditionally recognized for its role in motor coordination, has increasingly been acknowledged as a key brain region involved in a broad range of cognitive and emotional functions [33]. Recent studies have highlighted the cerebellum’s contribution to higher-order processes, extending its importance well beyond motor control [34]. In this context, *PTEN* dysregulation has been implicated in both neurodevelopmental disorders and neurodegenerative diseases that affect the cerebellar function [35]. Understanding the complex mechanisms by which *PTEN* influences cerebellar development and activity is essential for unraveling its role in various neurological conditions.

Previously, we established a systematic timeline and cellular landscape of human cerebellar organoid development, encompassing the differentiation of Purkinje cells, granule cells, interneurons, and astrocytes, and providing a foundation for the investigation developmental cerebellar disorders [36]. Building on this framework, we combine our cerebellar organoid model with iPSC cells carrying the *PTEN* Ile135Leu variant with mild loss of PTEN function that is found in an individual with ASD and macrocephaly, compared with a *PTEN* knockout [25, 32]. The goal was to examine how *PTEN* mutation or knockout as well as genetic background (using control or ASD iPSC models) affect cerebellar differentiation and development over 22-weeks of cerebellar organoid development. We found that *PTEN* Ile135Leu variant impaired cerebellar synaptic connectivity and neuronal circuitry, while *PTEN* knockout markedly altered the developmental trajectory-characterized by loss of the neuronal circuits and upregulation. Under both control and ASD-relevant genetic background, the *PTEN* Ile135Leu variant permitted the development of Purkinje cells, granule cells and interneurons, although this was significantly diminished or absent in the *PTEN* knockout.

## Results

### Temporal and spatial cerebellar populations in cerebellar organoids from isogenic *PTEN* panels of iPSCs in control and ASD backgrounds

We investigated the spatiotemporal cellular development of cerebellar organoids derived from our isogenic *PTEN* iPSC lines in both control (Chap) and ASD (Apex) genetic backgrounds. This included control wild type (Chap WT/WT), ASD *PTEN* wild-type (Apex WT/WT), control *PTEN* p.Ile135Leu variant (Chap WT/I135L), ASD *PTEN* p.Ile135Leu variant (Apex WT/I135L), control *PTEN* knockout (Chap KO/KO) and ASD *PTEN* knockout (Apex KO/KO) lines [32].

To assess early organoid growth, we quantified organoid size from day 1 to day 150, focusing on the period between 42 days and 100 days (Supplemental Fig. 1). Compared to their respective WT/WT counterparts, both Chap KO/KO and Apex KO/KO organoids exhibited significantly increased size after day 50 (D50). Notably, only the Apex WT/I135L organoids demonstrated enlargement after day 73 (D73), suggesting that the ASD-specific *PTEN* I135L variant affects organoid growth in a background-dependent manner, with no significant effect in the control background.

To evaluate cellular composition and differentiation over time, we performed immunohistochemical (IHC) staining and quantification at multiple time points (D35, D55, D80, D115, and D156), following our established protocol [36]. Cerebellar organoids were stained for Purkinje cells (KIRREL2, Calbindin D28K [CALB], PCP2), granule cells (ATOH1, PAX6, NEUROD1), interneurons (PAX2), synaptic markers (synaptophysin [SP]), and glial cells (GFAP, S100B). The Chap WT/WT line served as the baseline control and results were consistent with our prior results from this line [36].

**Figure 1:**
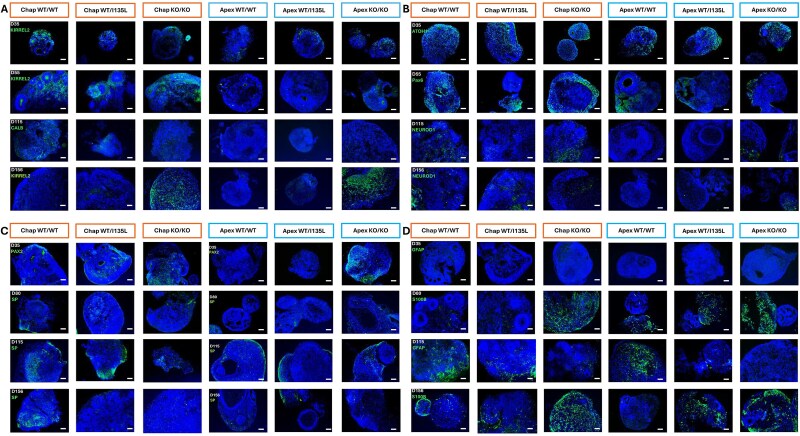
Isogenic *PTEN* panel iPSCs in control (Chap) background and ASD (Apex) background were differentiated and developed cerebellar organoids as experiment 2 (Exp2). Chap WT/WT refers to control line Chap with wild-type *PTEN*; Chap WT/I135L refers to control line Chap installed with the ASD heterozygous *PTEN* c.403A > C variant with CRISPR-Cas9; and Chap KO/KO denotes control line Chap with complete disruption of *PTEN*. Apex WT/WT refers to ASD line Apex with heterozygous *PTEN* c.403A > C variant corrected to wild-type *PTEN*; Apex WT/I135L refers to the original ASD line Apex, which contains heterozygous *PTEN* c. 403A > C variant; and Apex KO/KO refers to ASD line apex with complete disruption of *PTEN*. The whole cerebellar organoids were fixed and sectioned to investigate their spatiotemporal expression patterns at post-differentiation D35, D55, D80, D115, and D156. Immunohistochemistry (IHC) staining was performed using markers KIRREL2, CALB, PCP2 (A); ATOH1, PAX6, NEUROD1 (B); SP, PAX2 (C); and S100B, GFAP (D). Scale bar: 20 μm.

**Figure 2:**
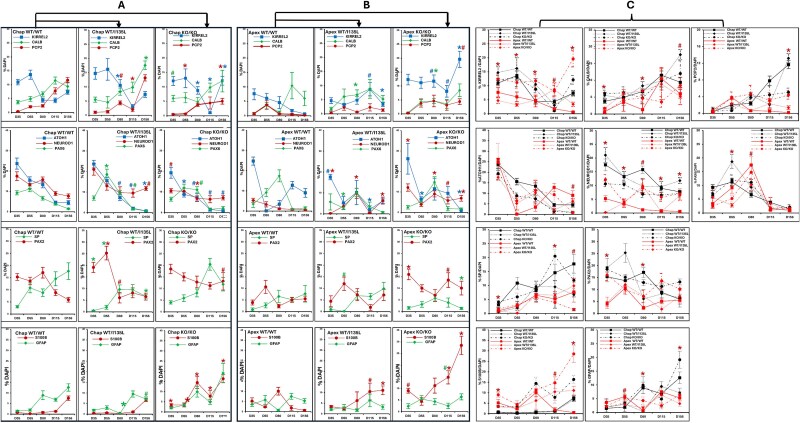
IHC data from Fig. 1 were quantified for the markers KIRREL2, CALB, PCP2 (PC); ATOH1, PAX6, NEUROD1 (GC); SP, PAX2 (IN); and S100B, GFAP (glial) in control (Chap) background (A) between Chap WT/WT to Chap WT/I135L and Chap KO/KO and in ASD (Apex) background (B) between Apex WT/WT to Apex WT/I135L and Apex KO/KO (n > 12) (two-tailed t-test, ^*^P < 0.01 and ^#^ P < 0.05, color matched the protein’s expression line). For direct visual comparison of the effects of both PTEN status and genetic background, each panel includes per marker was expressed from 35 days to 156 days displaying Chap WT/WT, Chap WT/I135L, Chap KO/KO in black and Apex WT/WT, Apex WT/I135L, Apex KO/KO in red (C) (*n* > 12) (ANOVA: Two-factor with replication, ^*^P < 0.01 and ^#^ P < 0.05, color and line style matched the protein’s expression line).

**In the control (Chap) background**, Chap WT/I135L organoids displayed significantly increased expression of mature Purkinje cells (CALB^+^) at D156, while PCP2^+^ cells were reduced at D115, compared with Chap WT/WT organoids (Figs 1A and 2A). In Chap KO/KO organoids, the progenitor marker KIRREL2^+^ cells were elevated at D156, while PCP2^+^ mature Purkinje cells decreased after D55, compared with Chap WT/WT organoids (Figs 1A and 2A). For granule cell development, Chap WT/I135L organoids exhibited a fewer ATOH1^+^ cells after D80 and an increased number of NEUROD1^+^ cells at D156, compared with their expressions in Chap WT/WT organoids (Figs 1B and 2A), indicating accelerated granule cell maturation. In contrast, Chap KO/KO organoids displayed significantly a reduced number of ATOH1^+^ and NEUROD1^+^ cells at D80, with sustained ATOH1^+^ cells’ reduction, thereafter, compared with cells in Chap WT/WT organoids. For interneurons, PAX2^+^ cells increased significantly cell numbers at D55 in Chap WT/I135L and at D156 in Chap KO/KO, compared with PAX2^+^ cells in Chap WT/WT organoids. SP^+^ cells were notably decreased in cellular number in Chap WT/I135L, compared with SP^+^ cells in Chap WT/WT organoids (Figs 1C and 2A). Glial cell analysis revealed reduced GFAP^+^ cells at D80 and D156 in Chap WT/I135L, whereas S100B^+^ cells were consistently elevated across all time points in Chap KO/KO organoids, compared with S100B^+^ cells in Chap WT/WT organoids (Figs 1D and 2A). These results demonstrate that, in the control (Chap) background, the *PTEN* I135L mutation increased the number of both mature Purkinje cells and granule cells at D156, while the proportion of SP^+^ cells decreased across almost all time points. In contrast, *PTEN* knockout leads to an increase in Purkinje progenitor cells and interneurons progenitor cells at D156, along with a sustained increase in astroglia cells from the onset of differentiation.

**In the ASD (Apex) genetic background**, both Apex WT/I135L and Apex KO/KO organoids displayed a higher proportion of KIRREL2^+^ progenitor Purkinje cells after D115 and elevated CALB^+^ cells at D80, compared cells to Apex WT/WT organoids. Notably, Apex KO/KO organoids exhibited enhanced proportion of PCP2^+^ mature Purkinje cells at D156 (Figs 1A and 2B). Regarding granule cell development, both Apex WT/I135L and Apex KO/KO lines demonstrated a higher proportion of NEUROD1^+^ cells and a steep decline in proportion of ATOH1^+^ cells after D115, suggesting accelerated granule cells maturation (Figs 1B and 2B). This pattern closely resembled that of *PTEN* WT/I135L in the control (Chap) background (Fig. 1B). PAX2^+^ cell numbers were elevated in Apex KO/KO at D35 and D115. SP^+^ cells number was significantly reduced in Apex KO/KO at D156 (Figs 1C and 2B), mirroring the reduction cells number observed in *PTEN* WT/I135L in the control (Chap) background (Figs 1C and 2A). GFAP^+^ cells number was decreased at D115 in Apex KO/KO organoids, while S100B^+^ cells number was elevated after D115 in both WT/I135L and KO/KO lines (Figs 1D and 2B), a pattern resembling that of *PTEN* KO/KO in the control (Chap) background (Figs 1D and 2A). These data indicate that, in the ASD (Apex) genetic background, the *PTEN* I135L mutation increased the number of Purkinje progenitor cells, mature granule cells, and astrocytes at and beyond D115. This aligns with our previous findings that *PTEN* WT/I135L promotes both neurogenesis and gliogenesis in cortical organoids [32]. In contrast, *PTEN* knockout led to an increase in both mature Purkinje cells and Purkinje progenitor cells at D156, along with enhanced granule cells maturation. Meanwhile, SP^+^ cells number was significantly decreased after D115. Additionally, *PTEN* knockout organoids exhibited an early and sustained increase in astroglia cells, like what is observed in the control (Chap) background.

In our previous study, we reported genetic background–dependent differences in neural progenitor cell development using cerebral organoid models [32]. While both the *PTEN* ASD variant and knockout influenced organoid size in a background-specific fashion, it remained unclear which cell types were most affected. To address this, we statistically compared cell type numbers in Chap and Apex organoids across all three genotypes (WT/WT, WT/I135L, KO/KO) using two-way ANOVA (Fig. 2C), then performed a series Bonferroni-corrected analyses of post-hoc t-tests (Table 1).

**Table 1:** Quantification of IHC on cerebellar organoids of each of the genotypes (WT/WT, WT/I135L and KO/KO) in either control or ASD genetic backgrounds from Fig. 2 over several time points (D35, D55, D80, D115 and/or D156) were subjected to two way ANOVAs based on genotype and genetic background with Bonferroni Correction as a post-hoc test for multiple testing reveals significant differences for several comparisons in markers from Purkinje cells (A, PC), granule cells (B, GC) as well as interneurons and glial cells (C, IN and GC). Significance is indicated by p values, while non-significant values are indicated by n.s.

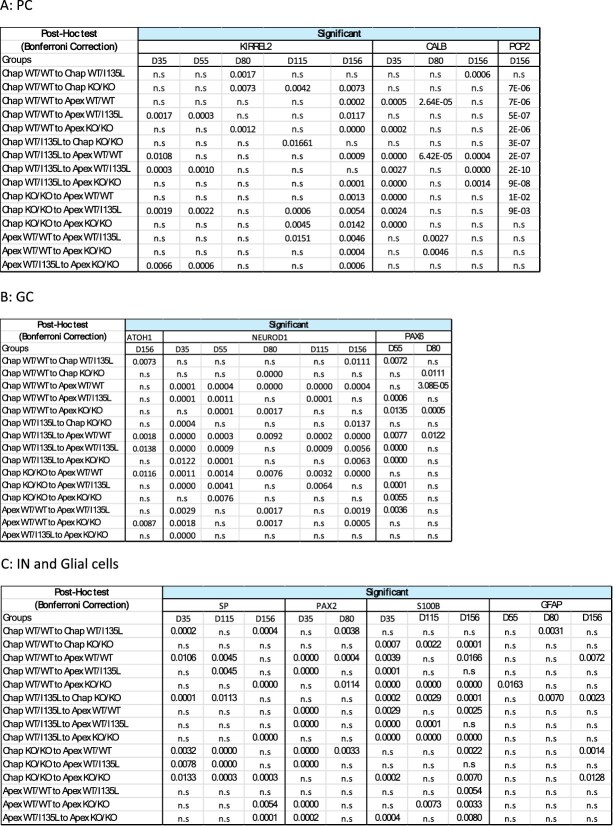

In ASD (Apex) background organoids, the proportion of CALB^+^ and PCP2^+^ (Purkinje cell markers), NEUROD1^+^ (a granule cell marker), and SP^+^ (synaptic marker), and GFAP^+^ (radial glia marker) cell numbers were significantly reduced after D115 compared to control (Chap) background organoids. Additionally, the proportion of KIRREL2^+^ (progenitor Purkinje marker) and ATOH1^+^ (Rhombic Lip marker) cells number was significantly increased after D115, indicating delayed cerebellar neuronal maturation (Fig. 2C). S100B^+^ cells number was markedly elevated during early developmental stages but declined after D115 in Apex WT/WT organoids, suggesting impaired or reduced glial development in Apex WT/WT organoids. At D156, Apex WT/I135L organoids had a lower proportion of KIRREL2^+^, CALB^+^, PCP2^+^, and NEUROD1^+^cells, but a higher proportion of S100B^+^ cells compared to Chap WT/I135L (Fig. 2C), indicating delayed Purkinje cells and granule cells differentiation alongside enhanced astrocyte development. In Apex KO/KO organoids, KIRREL2^+^ and S100B^+^ cells numbers were higher than cells numbers in Chap KO/KO, while the proportion of SP^+^ cells was reduced at D156 (Fig. 2C), suggesting that *PTEN* knockout in an ASD background alters the balance between neuronal and glial lineage commitment. This is consistent with our previous findings that Apex KO/KO promotes both neurogenesis and gliogenesis in cortical organoids compared with Chap KO/KO [32]. Our observations suggest that the differentiation and development of Purkinje cells, granule cells, and astrocyte differ between the control (Chap) and ASD (Apex) genetic background. However, these differences appear to be attenuated in the context of PTEN knockout.

We replicated these findings using Chap lines (Chap WT/WT, Chap WT/I135L, Chap KO/KO), which demonstrated that they were reproducible using the same IHC staining protocol (Supplemental Figs 2, 3). To further examine the re-emergence of Purkinje cell progenitor features, we analyzed the proportion of KIRREL2^+^ cells across D35–D156. Remarkably, KIRREL2^+^ Purkinje progenitors reappeared exclusively in Chap KO/KO, indicating that *PTEN* loss may induce neuroepithelial reprogramming regardless of genetic background (Supplemental Fig. 4A and C). Supporting this, NESTIN^+^ cellular expression—a neuroepithelial stem cell marker—was observed only in KO/KO organoids at D156 (Supplemental Fig. 4B), suggesting that Purkinje progenitors may re-enter the cell cycle under *PTEN*-deficient conditions.

To further assess development, we performed 3D immunostaining for KIRREL2^+^ and PAX6^+^ cells at D35 and D55 in Chap organoids. At D55, Chap WT/WT organoids exhibited defined apical-basal polarity, while WT/I135L organoids lacked such structural organization (Supplemental Fig. 5). Chap KO/KO organoids displayed severely disrupted morphology with ruptured structures and incomplete marker expression, displaying only about half the proportion of KIRREL2^+^ and PAX6^+^ cells (Supplemental Fig. 5), indicating that complete loss of *PTEN* may impair structural integrity of organoids and/or neuronal differentiation.

### Molecular studies of *PTEN* variants and genetic backgrounds in the cerebellar development

**Figure 3:**
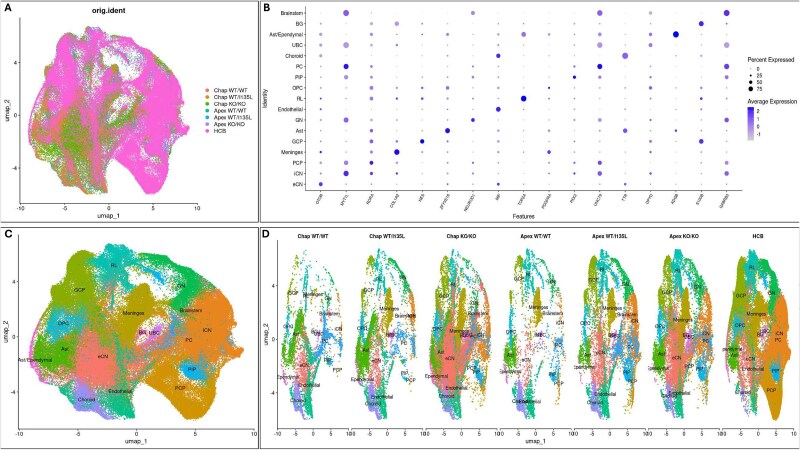
Comparison of cell types derived from isogenic *PTEN* panel cerebellar organoids with previously published dataset [37]. (A) UMAP plot of the six D55 libraries from our study were integrated with a published study of scRNA-seq datasets from human prenatal cerebellum from 9–22 postconceptional weeks (HCB) [37]. (B) DotPlot depicting gene expressions in different cell types. (C) Visualization of the same UMAP plot as in a, but color coded for the 17 classes of cell types found in B. (D) UMAP plots of each of the individual libraries with visualization of the same 17 classes of cell types found in B and C.

**Figure 4:**
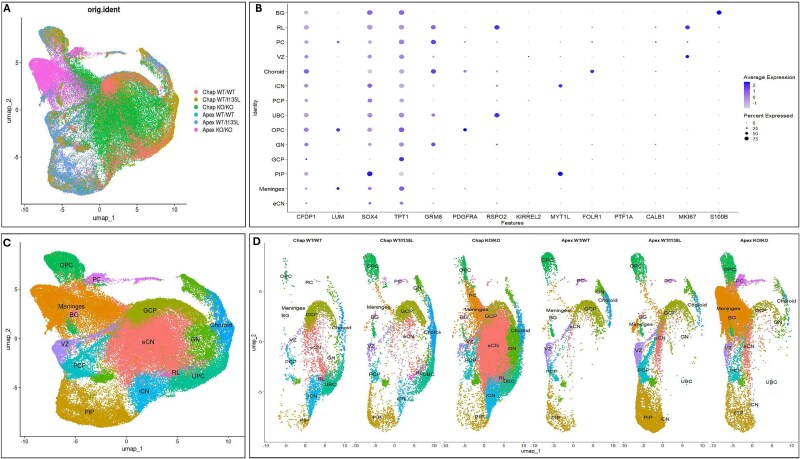
ScRNA-seq analysis of isogenic *PTEN* panel cerebellar organoids. (A) Identification of major cell types by combining the data from Chap WT/WT, Chap WT/I135L, Chap KO/KO, Apex WT/WT, Apex WT/I135L and Apex KO/KO organoids at D55 cerebellar organoids. UMAP visualization of cells from cerebellar organoids were colored for each individual data: Chap WT/WT (*n* = 6063), Chap WT/I135L (*n* = 8588), Chap KO/KO (*n* = 32 643), Apex WT/WT (*n* = 5220); Apex WT/I135L (*n* = 12 069) and Apex KO/KO (*n* = 19 281). (B) DotPlot depicting gene expressions in different cell types. (C) Visualization of the same UMAP plot as in a, but color coded for the 14 classes of cell types found in B. (D) UMAP plots of each of the individual libraries with visualization of the same 14 classes of cell types found in B and C.

To gain deeper insight into how *PTEN* influences cerebellar cell differentiation and development at the transcriptomic level, we performed single-cell RNA sequencing (scRNA-seq) on cerebellar organoids derived from six *PTEN* isogenic lines: Chap WT/WT, Chap WT/I135L, Chap KO/KO, Apex WT/WT, Apex WT/I135L, and Apex KO/KO. Organoids at D55 were analyzed using the 10X Genomics platform (Figs 3, 4), while organoids at D80 from Chap WT/WT, Chap WT/I135L, and Chap KO/KO were analyzed using the Drop-seq method [37] (Supplemental Fig. 6).

For the 10X scRNA-seq at D55, we obtained high-quality libraries containing: 6, 063 single cells from Chap WT/WT, 8, 588 from Chap WT/I135L, 32, 643 from Chap KO/KO, 5, 220 from Apex WT/WT, 12, 069 from Apex WT/I135L, and 19, 281 from Apex KO/KO. The corresponding gene counts per condition ranged from 29, 705 to 32, 132. For the Drop-seq at D80, the libraries included: 2, 081 single cells from Chap WT/WT, 836 from Chap WT/I135L, and 618 from Chap KO/KO, with gene counts of 17, 057, 22, 987, and 17, 425, respectively.

To assess the effects of *PTEN* mutation and knockout on cerebellar differentiation, we performed three complementary analyses: (1) integration with a published human cerebellum dataset spanning 9–22 post-conceptional weeks (HCB) [38] (Fig. 3); (2) merging of all six D55 libraries to identify the population of cell types in organoids (Fig. 4); and (3) comparison of gene expression patterns among genotypes and genetic backgrounds by gene set enrichment analysis (GSEA). A unified UMAP plot incorporating all six D55 scRNA-seq libraries revealed 14 transcriptionally distinct clusters corresponding to cerebellar cell types, annotated using canonical markers [36] (Fig. 4C). These included Bergmann glial (BG), rhombic lip (RL), Purkinje cells (PC), ventricular zone (VZ), choroid plexus (Choroid), inhibitory cerebellar nuclei (iCN), Purkinje cell progenitors (PCP), unipolar brush cells (UBC), oligodendrocyte precursor cells (OPC), endothelial cells, granule cell progenitors (GCP), interneuron progenitors (PIP), meningeal cells (Meninges), and excitatory cerebellar nuclei neurons (eCN) (Fig. 4B). The individual separated UMAP from all libraries (Fig. 4D) confirmed that our cerebellar organoids recapitulate major human cerebellar cell types.

**Figure 5:**
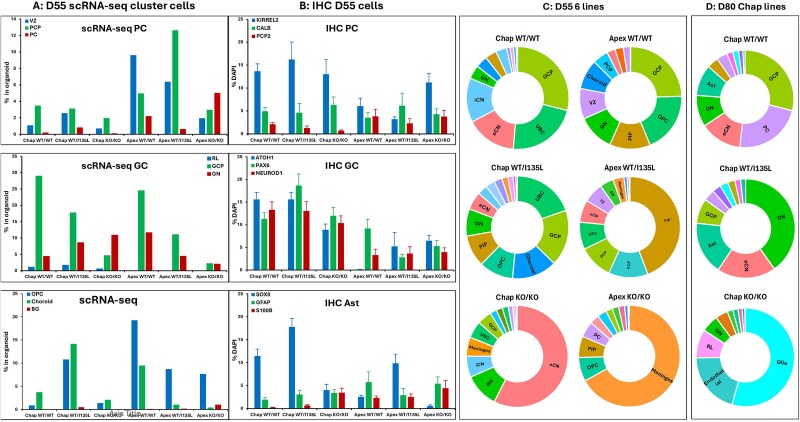
Comparing the cellular population in organoids between methods in IHC and scRNA-seq. (A) Purkinje cells (vz-KIRREL2, PCP-KIRREL2-CALB, PC-PCP2), granule cells (RL-ATOH1, GCP-ATOH1/PAX6, GN-NEUROD1) and astrocyte (ORC-SOX9, BG-S100B) population in organoids at D55 from Chap WT/WT, Chap WT/I135L, Chap KO/KO, Apex WT/WT, Apex WT/I135L and Apex KO/KO. (B) IHC 2D stained with neurons and astrocytes antibodies. (C) Sunburst plots of all cellular population at D55 from six isogenic *PTEN* panel cerebellar organoids in control and ASD genetic background. (D) Sunburst plots of all cellular population at D80 from three isogeneic PTEN panel cerebellar organoids in control background.

Cell type composition was identified through marker gene analysis (Figs 3B and 4B), and differences in composition across genotypes and backgrounds were visualized using individual UMAP plots (Figs 3D and 4D). Individual UMAPs (Fig. 4D) revealed elevated OPC and PIP populations in both backgrounds of *PTEN* WT/I135L and *PTEN* KO/KO lines. At D55, Apex KO/KO organoids were enriched in meningeal cells, while Chap KO/KO organoids were enriched in eCN neurons. Quantitatively, GCPs constituted: 28.95% in Chap WT/WT; 17.70% in Chap WT/I135L; 4.62% in Chap KO/KO; 24.48% in Apex WT/WT; 11.01% in Apex WT/I135L; and 2.14% in Apex KO/KO (Fig. 5A). This distribution aligns with IHC quantification of ATOH1 and PAX6 (Fig. 5B). In addition, the similarity of GCP percentages between control (Chap) and ASD (Apex) backgrounds suggests that the *PTEN* genotype is the principal determinant of GCP differentiation.

Remarkably, Chap KO/KO organoids contained 57.45% eCNs, while PIPs dominated Apex WT/I135L organoids (44.20%), and meningeal cells comprised 66.90% of Apex KO/KO cells (Fig. 5C). These skewed distributions of PIP in Apex WT/I135L may help explain previous findings—including our MEA data (not shown) and those reported by Marchetto et al., 2017 [25]—suggesting that Apex WT/I135L neurons lose neuronal communication due to unbalanced differentiation of inhibitory neurons. *PTEN* knockout showed an early and sustained increase in astroglia cells in both Chap KO/KO and Apex KO/KO organoids (Fig. 2C), combined with accelerating meningeal-like cells development in Chap KO/KO and Apex KO/KO organoids.

At D80, Drop-seq analysis further confirmed divergent developmental distributions. A UMAP revealed that cell distributions from Chap WT/WT (WT/WT) and Chap WT/I135L (*PTEN* WT/I135L) organoids largely overlapped (Supplemental Fig. 6A), while cell distributions from Chap KO/KO (*PTEN* KO/KO) organoids clustered separately (Supplemental Fig. 6A and E). The dominant cell types at this stage in Chap WT/WT were PC, GCP, CN and PIPs, but Chap KO/KO organoids displayed marked enrichment of glial and endothelial populations (Supplemental Fig. 6E), supporting IHC results of elevated GFAP and S100B (Figs 1 and 2A and Supplemental Fig. 2).

**Figure 6:**
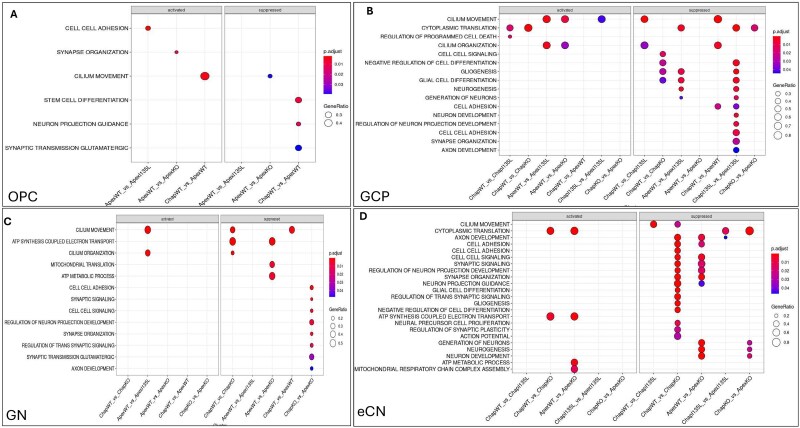
GSEA for the effect of glutamatergic neurons in the control background, ASD genetic background and between same mutation from Chap and Apex isogenic *PTEN* panel in D55 cerebellar organoids. OPC (A), GCP (B), GN (C), eCN (D).

**Figure 7:**
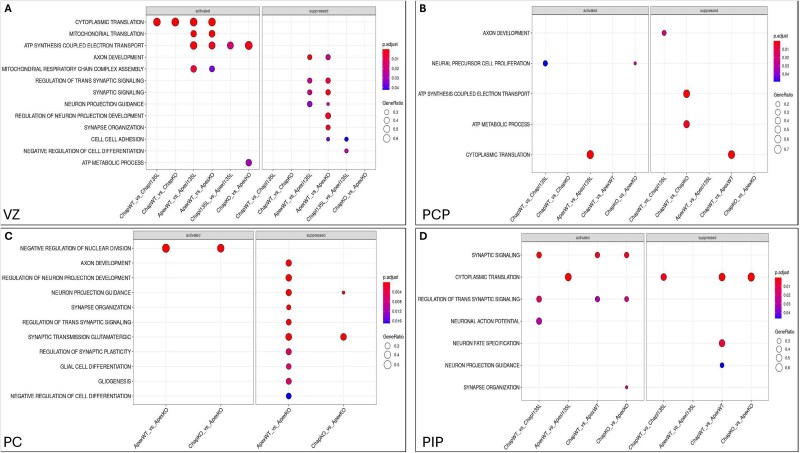
GSEA for the effect of GABAergic neurons in the control background, ASD genetic background and between same mutation from Chap and Apex isogenic *PTEN* panel in D55 cerebellar organoids. VZ (A), PCP (B), PC(C), PIP (D).

Finally, GSEA was performed to uncover unbiased gene expression shifts across genotypes and genetic backgrounds. We compared: Chap WT/WT vs. Chap WT/I135L, Chap WT/WT vs. Chap KO/KO, Apex WT/WT vs. Apex WT/I135L, Apex WT/WT vs. Apex KO/KO, Chap WT/WT vs. Apex WT/WT, Chap WT/I135L vs. Apex WT/I135L, and Chap KO/KO vs. Apex KO/KO. Enriched GO terms across these comparisons highlighted processes such as neurogenesis, gliogenesis, synaptic signaling, apoptosis, cilium movement, and metabolism—key pathways in cerebellar glutamatergic and GABAergic neuron development (Figs 6 and 7 and Supplemental Figs 9–18). For example, compared to Chap WT/WT, Apex WT/WT organoids displayed upregulation of OPC stem cell differentiation and neuron projection guidance at D55 (Fig. 6A), suggesting that ASD-related background modulates *PTEN*-dependent cell fate specification.

### GSEA in glutamatergic neuronal differentiation and development

In the control (Chap) background, the *PTEN* ASD variant (Chap WT/I135L) altered granule cell progenitor (GCP) behavior by downregulating apoptosis and upregulating cilium movement—two critical processes for sustaining the GCP pool and promoting granule neuron differentiation. Conversely, in the ASD (Apex) background, both *PTEN* WT/I135L (Apex WT/I135L) and KO/KO (Apex KO/KO) lines displayed downregulation of cilium movement and cilium organization in GCPs, indicating disruption in GCP homeostasis.

When comparing Apex WT/WT to Chap WT/WT, Apex WT/WT organoids exhibited upregulation of pathway associated with cilium movement and cell adhesion, both of which are critical for neuroepithelial organization. At the level of oligodendrocyte precursor cells (OPC), Apex WT/WT organoids also exhibited increased expression of genes involved in stem cell differentiation, neuron projection guidance, and glutamatergic synaptic transmission (Fig. 6A). Interestingly, the ASD variant in Apex (Apex WT/I135L), but not in Chap (Chap WT/I135L), upregulated pathways related to neurogenesis and granule neuron development, underscoring the background-specific effects of the *PTEN* I135L mutation on GCP differentiation (Fig. 6B).

Further comparisons in the *PTEN* WT/I135L context revealed that the ASD variant in the ASD background (Apex WT/I135L) enhanced gene programs for neurogenesis, neuronal differentiation, gliogenesis, glial cell development, and axon development (Fig. 6B). These findings are consistent with our previous results from cortical organoids and NPC differentiation using the same iPSC lines [32].

At the level of mature granule neurons (GNs), Apex WT/WT organoids displayed upregulation of cilium movement compared to Chap WT/WT (Fig. 6C), reinforcing the idea that *PTEN* function impacts ciliary and developmental signaling pathways. In excitatory cerebellar nuclei (eCN) neurons, *PTEN* KO/KO organoids in the control background (Chap KO/KO) upregulated gliogenesis and neural precursor cell proliferation, while in the ASD background (Apex KO/KO), *PTEN* deficiency led to enhanced neurogenesis and neuron development in eCNs (Fig. 6D). These findings agree with our IHC data demonstrating elevated S100B expression in Chap KO/KO organoids (Figs 1D, 2A and C), further indicating increased gliogenic activity in both GCP and eCN lineages after *PTEN* loss. Overall, these results provided evidence that both *PTEN* mutation and *PTEN* knockout regulated neurogenesis, glicogenesis, cilium organization and cell adhesion during glutamatergic neuronal differentiation and development in both control and ASD genetic backgrounds related with different neuronal populations.

### GSEA in GABAergic neuronal differentiation and development

In the ASD background, the ASD variant (Apex WT/I135L and Apex KO/KO) induced strong upregulation of axon development, synaptic signaling, and neuron projection guidance in ventricular zone (VZ) cells. Cross-background comparison of WT/I135L lines revealed that this variant also enhanced negative regulation of VZ cell differentiation and upregulated cell–cell adhesion, suggesting disrupted progenitor maintenance and neuroepithelial architecture (Fig. 7A).

In Purkinje cell precursors (PCPs), the *PTEN* WT/I135L genotype in the control background (Chap WT/I135L) led to reduced proliferation and increased axon development (Fig. 7B). In the ASD background, the *PTEN* KO/KO genotype (Apex KO/KO) further amplified axonogenesis and promoted Purkinje neuron projection (Fig. 7C), which was consistent with increased KIRREL2+ cells observed via IHC in day 80 organoids (Fig. 1A, Fig. 2A-C).

For interneuron progenitors (PIPs), significantly upregulated pathways involved in interneuron fate commitment and neuron projection guidance were found in WT/WT in the ASD background (Apex WT/WT, Fig. 7D). Conversely, downregulated synaptic signaling, trans-synaptic signaling regulation, and neuronal action potential were found in *PTEN* WT/I135L in the control background (Chap WT/I135L), suggesting impaired functional maturation and communication between neurons in Chap WT/I135L versus Chap WT/WT organoids.

### *PTEN* mutation affects neuronal communication

Previous studies have demonstrated that *PTEN* plays a critical role in embryonic neurogenesis, particularly through the regulation of neural progenitor cell (NPC) proliferation. In our earlier work using Apex WT/I135L NPCs and cerebellar organoids, we observed few neuronal spikes and no measurable neuronal communication [25]. In contrast, Chap cerebellar organoids exhibited active neuronal communication, as confirmed by electrophysiological measurements and supported by single-cell RNA sequencing (scRNA-seq), which revealed that the *PTEN* p.Ile135Leu variant disrupts genes involved in neurogenesis, neuronal development, and synaptic signaling [36].

To further investigate *PTEN*’s role in established neuronal networks, we assessed the effect of the *PTEN* p.Ile135Leu variant (WT/I135L) on synaptic signaling using multi-electrode arrays (MEAs), a platform that enables real-time recording of neuronal activity from multiple electrodes simultaneously [39]. We analyzed Day 80 cerebellar organoids from six genotypic groups: Chap WT/WT (A1–A8), Chap WT/I135L (B1–B8), Chap KO/KO (C1–C8), Apex WT/WT (F1–F8), Apex WT/I135L (D1–D8), Apex KO/KO (E1–E8). Each organoid was loaded into an individual well of a 48-well MEA plate, ensuring central positioning to maximize contact with 16 embedded platinum microelectrodes per well (50 μm diameter, 350 μm spacing), totaling 384 channels. Heatmaps and raster plots were generated to visualize activity from representative organoids [36].

**Figure 8:**
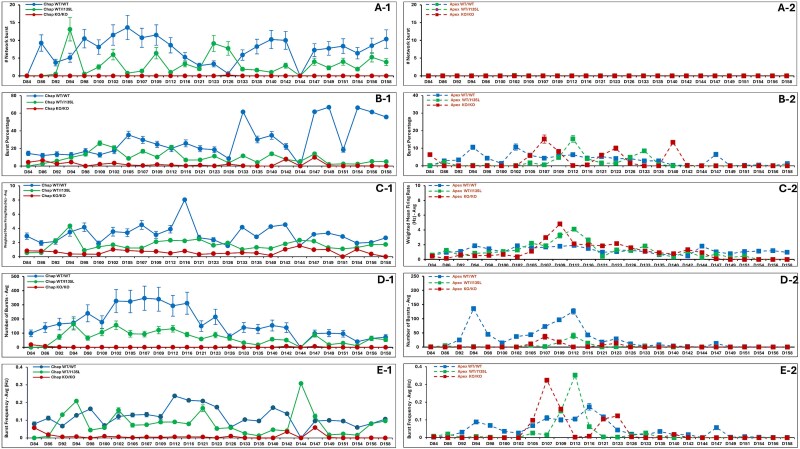
Neuronal communication was generated with neural metric tool v3.5.1 software (Axion biosystems) and recorded from 84 days (D84) to 158 days (D158) cerebellar organoids (8 organoids/group). The key metrics included the number of network bursts from control background (Fig. 9A-1) and from ASD genetic background (Fig. 9A-2), burst percentage from control background (Fig. 9B-1) and from ASD genetic background (Fig. 9B-2), weighted mean firing rate from control background (Fig. 9C-1) and from ASD genetic background (Fig. 9C-2), total burst count from control background (Fig. 9D-1) and from ASD genetic background (Fig. 9D-2), and burst frequency from control background (Fig. 9E-1) and from ASD genetic background (Fig. 9E-2).

To quantify neuronal activity, we recorded from eight organoids per group, analyzing data across 16 electrodes per organoid. Metrics included: Number of network bursts (Fig. 8A); Burst percentage (Fig. 8B); Weighted mean firing rate (Fig. 8C); Total burst count (Fig. 8D); and Burst frequency (Fig. 8E). Recordings from day 84 to day 158 revealed that only Chap WT/WT and Chap WT/I135L consistently formed functional neuronal networks. Among Apex organoids, Apex WT/WT organoids displayed intermittent bursting prior to day 123 (D123).

**Figure 9:**
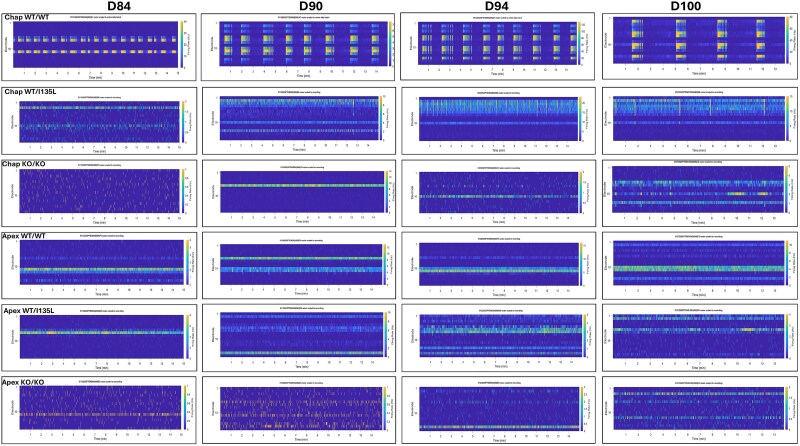
Neuronal communication was generated with MEA-NAP software and raster plots from Chap WT/WT organoid (A7), Chap WT/I135L (B6), Chap KO/KO (C5), Apex WT/WT (F5), Apex WT/I135L (D8) and Apex KO/KO (E5) at D84, D90, D94 and D100.

**Figure 10:**
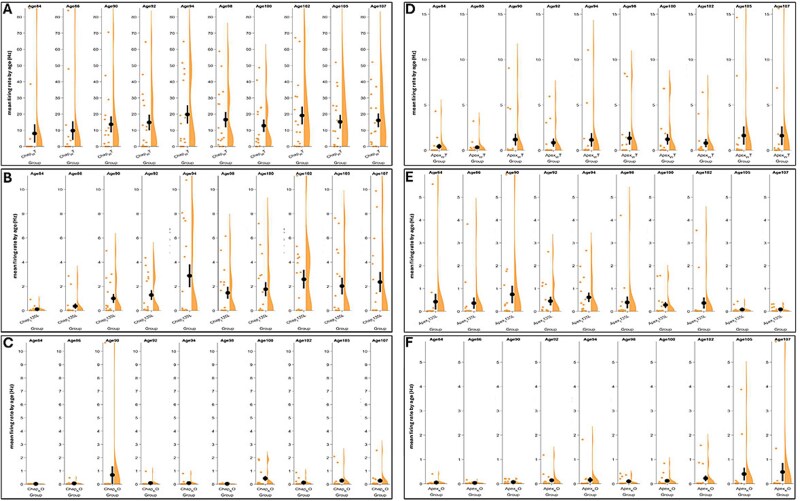
Neuronal communication of mean firing rate (Hz) was generated with MEA-NAP software from D84 (Age84) to D107 (Age107) from Chap WT/WT (A), Chap WT/I135L (B), Chap KO/KO (C), Apex WT/WT (D), Apex WT/I135L (E), and Apex KO/KO (F).

We applied MEA–Network Activity Profiling (MEA-NAP) method to assess network connectivity and microscale dynamics in each group of organoids [40]. In the MEA-NAP analysis, an active organoid from each group (A7, B6, C5, F5, D8, E5) served as a representative sample. The raster plots depicted spike trains recorded from MEA recording for 15 min from D84 to D100 (Fig. 9). To identify the neuronal subtypes driving spontaneous communication, we focused on Purkinje cells, the principal output neurons of the cerebellar cortex. Purkinje cells are unique in their intrinsic spontaneous firing patterns, which reflect cerebellar differentiation and maturation. In vivo studies in rats have shown that mature Purkinje cells exhibit two types of regenerative electrical behavior: Complex spikes (~1 Hz, range: 0.01–1.2 Hz) input from climbing fiber [41]; and simple spikes (~38.8 Hz, range: 20–100 Hz) spontaneous action potentials [39, 42]. We analyzed spike frequency in cerebellar organoids from Day 84 to Day 107 (Fig. 10A–F). While all genotypes exhibited low-frequency complex spikes, only Chap WT/WT organoids demonstrated consistent high-frequency simple spikes, indicative of functionally active and maturing Purkinje cells (Fig. 10A). This suggests that Chap WT/WT organoids most effectively recapitulate Purkinje cell development and maturation, compared to all other genotypes. Importantly, both the *PTEN* point mutation and *PTEN* knockout impaired simple spike generation in Purkinje cells—even in *PTEN*-corrected Apex WT/WT lines—highlighting a critical role for *PTEN* in functional Purkinje neuron development. Collectively, MEA recording in the control (Chap) background demonstrated that Chap WT/WT cerebellar organoids exhibited spontaneous activity driven by Purkinje cells, consistent with previous findings [36]. Although Chap WT/I135L organoids displayed similar patterns of neuronal network activity, the firing spikes did not reach the frequency of Purkinje cellular simple spikes, which was consistent with the finding that Chap WT/I135L induced downregulation of neurogenesis and neuronal development in Purkinje cells on D80 (Supplemental Fig. 8). In contrast, *PTEN* KO/KO organoids completely lacked spontaneous neuronal signaling (Fig. 10C and F). Gene set enrichment analysis (GSEA) of *PTEN* KO/KO organoids revealed upregulation of pathways related to gliogenesis and glial cell differentiation (Fig. 7C), which may contribute to the absence of spontaneous signaling. In the ASD (Apex) genetic background, MEA data demonstrated reduced spike activity, bursting, and network connectivity, indicating impaired synaptic maturation. These findings are consistent with immunocytochemistry results showing reduced expression of mature Purkinje cells markers and synaptophysin in the ASD (Apex) genetic background cell lines (Figs 1A and 2B).

## Discussion

Autism spectrum disorders (ASD) in humans are heterogeneous genetic conditions arising from a complex interplay of genetic and epigenetic factors. ASD likely arises from the complex interplay of genetic, epigenetic, and environmental factors during brain development. A major difficulty in deciphering the pathophysiology of ASD stems from its extensive genetic heterogeneity—over 100 genes have been implicated in its pathology. In addition, each individual with ASD brings a unique combination of genetic backgrounds. We and others have used human isogenic induced pluripotent stem cell (iPSC) models of cerebral development with controlled mutations and genetic backgrounds, to determine that both the mutation of a particular ASD gene and genetic background contribute to cellular phenotypes relevant to early brain overgrowth [25, 32, 43, 44].

In addition to the cerebral cortex, recent studies have established that the cerebellum plays a crucial role in cognitive and emotional processing, implicating cerebellar structure and function in ASD [45, 46], highlighting the involvement of Purkinje cells, granule cells, and/or glial cells in ASD-related phenotypes. The use of cerebellar organoids provides a powerful model to study how *PTEN*-induced abnormalities influence cerebellar structure and function, offering insight into the developmental mechanisms at play. Building on our previous studies on cerebellar development and differentiation using iPSCs, we optimized the quality and reproducibility of cerebellar organoids and investigated the effects of an ASD specific *PTEN* mutation or inactivation. We performed a parallel analysis of *PTEN* knockout lines in both control and ASD genetic backgrounds to examine the consequences of *PTEN* loss on cerebellar differentiation and development. Our findings highlight the potential of these organoids as a platform for studying cerebellar disorders and dissecting contributions of gene mutations and genetic background.

In this study, we found that the *PTEN* WT/I135L variant in an ASD individual exhibited distinct effects depending on the genetic background—either control or ASD—a finding that is consistent with our previous study in cerebral organoids [32]. When present in the control (Chap) background, the *PTEN* WT/I135L variant downregulated Purkinje cell progenitors (PCP) genes related to brain regionalization and mitotic nuclear division (Supplemental Fig. 13), correlating with enhanced expression of mature Purkinje and granule cells as shown by IHC analysis of Chap WT/I135L organoids. While MEA data revealed that these changes facilitated neuronal network formation, the resulting networks exhibited unstable neural communication compared to the Chap WT/WT line.

In the ASD (Apex) background, the variant also increased mature granule cells, along with elevated expression of ventricular zone marker KIRREL2 and astrocytic marker S100B. GSEA analysis supported the observation that the variant upregulated both neurogenesis and gliogenesis, particularly pathways related to neuronal generation and glial differentiation. Although the corrected ASD line (Apex WT/WT) displayed partial restoration of neuronal communication, MEA recordings indicated that the resulting bursts were insufficient to establish a stable functional network.

Organoids lacking *PTEN* were significantly larger, reflecting increased proliferation as evidenced by organoid size (Supplemental Fig. 1) and a higher yield of dissociated cells for scRNA-seq. In the absence of *PTEN*, differentiation distributions became biased toward specific lineages: excitatory cerebellar nuclei (eCN) neurons at day 55 and glial at day 80 in the control (Chap) background; and meninges-like cells at day 55 in the ASD (Apex) background (Fig. 6C and D). On day 156, IHC data showed similar outcomes across both backgrounds, with increased expression of KIRREL2, GFAP, and S100B (Supplemental Fig. 7). GSEA analysis further revealed enhanced granule cell progenitor (GCP) gliogenesis and glial differentiation in Chap KO/KO organoids with the control (Chap) background, and upregulated inhibitory cerebellar nuclei (iCN) neurogenesis and neuronal development in the ASD (Apex) background. Although gene sets related to eCN neuron projection guidance, synapse organization, and axon development were enriched in both backgrounds, no functional neuronal networks were detected before day 158. Among sixteen *PTEN*-KO organoids studied, only one displayed a stable and well-formed network. Notably, *PTEN* knockout produced similar patterns in IHC, scRNA-seq, and MEA data, although the affected cell populations differed at the transcriptomic level.

Despite the successful generation of cerebellar-like tissue, it must be noted that cerebellar organoid protocols are not as well established as those for cerebral organoids. Although our cerebellar organoid protocol resulted in the production of many of the cell types found in human cerebellar development, the organoids did not fully recapitulate the complex laminar organization observed in the developing human cerebellum.

This study offers new insights into the pathogenesis of *PTEN*-related ASD, using autism-specific iPSCs carrying the *PTEN* p.Ile135Leu variant and *PTEN* KO/KO lines. Our findings further validate cerebellar organoids as a robust and reproducible model system for investigating ASD-associated cerebellar dysfunction and for the development of personalized therapeutic strategies.

## Materials and methods

### Human iPSCs

All human iPSCs have been described in our previous studies. The control iPSC Chap (WT/WT) and the ASD patient derived iPSC cell line Apex (WT/I135L) [25] were used as parental cell lines and then modified to generate isogenic *PTEN* iPSC panels: *PTEN* WT/Ile135Leu and complete disruption of *PTEN* iPSC panels (*PTEN* KO/KO) in the control and ASD genetic backgrounds as described [32]. These lines are designated: Chap WT/WT, Chap WT/I135L, and Chap KO/KO for the control background; and Apex WT/WT, Apex WT/I135L and Apex KO/KO for the ASD genetic background.

### Cerebellar organoids

The original protocol for differentiating cerebellar organoids was described in a previous study [47], which we modified [36]. iPSC cells were cultured on a feeder layer of iMEF cells in hES medium consisting of DMEM/F12 (Gibco;11 330–032), 20% knockout serum replacement (Gibco; 10 828–028), 1% GlutaMAX (Gibco; 35 050–061), and 1% Non-essential AA (Gibco; 11 140–050). Prior to the experiment, ReleSR media (StemCell Technologies; 5872) was used to remove iMEF cells. Subsequently, iPSC cells were plated onto vitronectin-coated culture dishes (Life Technology; A31804) and cultured in E8 medium (Life Technology; A1517001) until they reached approximately 60% confluence. The iPSC cells were then collected and dissociated into single cells using TrypLE (Thermo Fisher; 12 605 093).

Next, cells were plated at a density of 6x10^3^ cells per well of V-shaped 96-well plates in CDM medium containing DMEM/F12 (Gibco:11330–032), 250 mg/ml BSA (Sigma: A2153), 15 μg/ml apo-transferrin (Sigma: T1147), 39 nl/ml 1-thioglycerol (Sigma: M6145), and 7 μg/ml insulin (Sigma: I2643). On the first day, half of the CDM medium was replaced with fresh medium containing 50 ng/ml βFgf2 (GoldBio: 1140-02-1000) and 10 μM SB431542 (ED millopore: 61646). On day 7 of the differentiation protocol, fresh CDM medium containing 25 ng/ml βFgf2 and 5 μM SB431542 was added to the cells. On day 14, the organoids were transferred from the V-shaped 96-well plate to a U-shaped 96-well plate containing a new CDM medium supplemented with 100 ng/ml Fgf19 (R&D system: 969-FG-025). On day 21, the culture medium was switched to CMM1 medium, which contained neurobasal medium (Thermo Fisher; 21 103 049), N2 supplement (Thermo Fisher; 17 502 048), and 1% Penicillin–streptomycin. On day 28, fresh CMM1 medium containing 300 ng/ml recombinant human SDF-1α (CXCL12) (Pepro TECH: 300-28A) was added. From day 35 onwards, all organoids were maintained in CMM1 medium.

### 2D immunocytochemistry

At specified intervals following the initiation of the organoids (D35, D55, D80, D115, and D156), the organoids were fixed using 4% (wt/vol) paraformaldehyde in Tris-Buffered Saline (TBS) for 1 hour, followed by washing with PBS, and subsequent submersion in TBS with 30% sucrose overnight. Afterward, the organoids were mounted in 50% OCT embedding compound and 50% of 30% sucrose, then frozen in dry ice and stored at −80°C.

For the preparation of organoid sections for staining, slices with a thickness of 20 μm were cut using a cryostat and thaw-mounted onto negatively charged histological slides. These slices were stored at −20°C for up to six months. Prior to staining with antibodies, the slides were allowed to thaw at room temperature for 10 min and then submerged in TBS for 5 min.

Antigen retrieval was performed using 10 mM sodium citrate and 0.05% Triton X-100 (pH 7.4), twice for 10 min each. To block nonspecific binding, 5% donkey serum in TBS was applied for 1 hour at room temperature. Primary antibodies were then applied overnight at 4°C, followed by secondary antibodies for 45 min at room temperature.

### 3D immunocytochemistry

To prepare the organoids for staining, we followed the same fixation process as outlined in the 2D immunocytochemistry protocol. Following fixation, the organoids were immersed in TBS with 30% sucrose overnight, then transferred to FG medium containing 60% glycerol and 2.5 M fructose for clearing. For staining, the entire organoid was incubated with the primary antibody for 24–48 h at 4°C on a shaker, followed by incubation with the secondary antibody for 24 h at 4°C on a shaker. DAPI staining was performed for 30 min at room temperature on a shaker.

Subsequently, the whole organoids were placed onto negatively charged histological slides, covered with mounting medium, gently overlaid with coverslips, and allowed to dry overnight at 4°C before imaging. Confocal microscopy was conducted using a Leica TCS SP8 gated STED 3X microscope equipped with a 10X objective, a numerical aperture of 0.4, and z-sectioned at 2 μm intervals.

### scRNA-seq library preparation and sequencing

To prepare the RNA libraries, we used two methods: 10x scRNA-seq (10x Genomics) and Drop-seq [37]. We used the same organoid dissociation protocol to obtain single-cell suspensions using the Papain kit (Worthington Biochemical Co. LK003153).

For 10X scRNA-seq, we used the Chromium Nest GEM Single Cell 3’ Kit v3.1 (10x Genomics, 1 000 268) for all scRNA-seq library preparations. Single-cell suspensions from Chap WT/WT, Chap WT/I135L, Chap KO/KO, Apex WT/WT. Apex WT/I135L and Apex KO/KO were loaded onto the chromium Next Chip G Single Cell Kit (10x Genomics, 1 000 120) and processed with Chromium Controller 10v Genomics (10x Genomics) to generate single-cell gel beads in emulsion. The libraries were sequenced on the NovaSeqX platform using paired-end sequencing, with the first read having a length of 28 bases and the second read having a length of 90 bases. Raw fastq files were uploaded into the 10x Genomics cloud and reads were aligned to Human (GRCh38) with Cell Ranger. The generated filtered raw matrix including barcodes, features and matrix were uploaded into Seurat v5, and LogNormalize was used for normalization.

In the Drop-seq protocol, we used the published protocol developed by the McCarroll laboratory, which is available online [37]. Briefly, we utilized barcoded beads (ChemGenes Corporation, Macosko-2011-10(V+)) suspended at 120, 000 beads/ml in Drop-seq lysis buffer comprising 200 mM Tris pH 7.5, 6% Ficoll PM-400, 0.2% Sarkosyl, 20 mM EDTA, and 50 mM DTT. Single-cell suspensions from Chap WT/WT, Chap WT/I135L and Chap KO/KO were suspended in PBS with 0.01% BSA at 0.2x10^6^ cells/ml. For droplet generation, we injected the cells, beads, and oil (Bio-Rad, 186–4006) at flow rates of 2 mL/h, 2 mL/h, and 7.5 mL/h, respectively, using syringe pumps. After droplet generation, we broke the droplets, washed, and collected the beads. The beads underwent reverse transcription using TSO_RNA hybrid primer, followed by exonuclease I digestion. The libraries were multiplexed and sequenced on a HiSeqX PE150 (Medgenome) to generate approximately 200 million total paired reads per library in gzip-compressed FASTQ files. We employed the Cell Ranger pipeline to align the reads from RNA-seq to the GRCh38 human reference genome and produce the associated cell-by-gene count matrix. Then, the matrix was uploaded into Seurat v5, and LogNormalize was used for normalization.

Data processing was conducted using the Seurat pipeline (satijalab.org). Cells were filtered based on unique feature counts (>2, 500 and < 200) and mitochondrial gene content (>5%). After normalization, dimensionality reduction was performed via principal component analysis (PCA), using a PCA of 50. Additionally, genes detected in fewer than 3 cells were removed. Finally, we used the Seurat function to partition the cells into transcriptionally distinct clusters (resolution 0.3) and presented the graph clustering output on a 2D map using UMAP. All filtered libraries were ranked based on the genes for Gene Set Enrichment Analysis (GSEA) using avg_log2 FC. We performed GSEA between genotypes by using clusterprofrofiler 4.5.1. GSEA was performed with the Molecular Signatures Database (msigdbr), using the function biological Process (BP) in the following link: GSEA | MSigDB | Human MSigDB Collections (gsea-msigdb.org).

hs_gsea_c5 < − msigdbr(species = ‘*Homo sapiens*’,

collection = ‘C5’,

subcategory = ‘BP’) % > %.

We used the compare cluster function to visualize the top 10 Gene Ontology (GO) term enrichments (Supplemental Figs 9–18) and to visualize across any function related to neurogenesis, gliogenesis, synaptic function and cilium movement (Figs 7 and 8) with default settings [48].

The gene expression data were deposited in Gene Expression Omnibus (GEO, accession number GSE310490).

### Multi-electrode array (MEA) preparation, recording and data analysis

To prepare the 48-well MEA plate (Axion Biosystems), we followed a published protocol [36, 49]. Briefly, the plate was coated with 100 μg/ml poly-L ornithine overnight, dried in the hood, rinsed three times with PBS, and then 300 μL of 10 μg/ml laminin (Sigma, 2020) in PBS was added to each well for 1 hour in the incubator before loading the organoids.

For each experiment, D80 organoids were loaded into each well. Prior to this MEA experiment, some organoids at D35 and D55 were stained with both 2D and 3D IHC to confirm differentiation. After one week, the plate was ready for recording. Neuronal communication was tracked over 15 min at 37°C and 5% CO_2_, every other day, for up to 250 days in this study. Data recording was conducted using the Maestro MEA system (Axion Biosystems). The spike detection threshold was set at 5 times the standard deviation using the Axis software. To begin recording, the plate was moved from the incubator and allowed to equilibrate in the Maestro device for five minutes. Burst and network analyses were performed using the Axis software. According to Axion Biosystem’s Neural Metris Tool, electrodes exhibiting at least five spikes per minute as were classified as active. Bursts were defined by an interspike interval (ISI) threshold, requiring a minimum of 5 spikes and a maximum ISI of 100 ms. The MEA-NAP software [40] was used to analyze detected spikes frequency from individual electrons and distinct network topologies.

### Statistical analysis

IHC and MEA data were presented as mean ± SEM, whereas all other data were presented as mean ± SD. Statistical significance was evaluated using two-tailed t-test for pairwise comparisons, two-factor ANOVA with replication to assess the effects of PTEN status and background at different time points and post-hoc tests (Bonferroni Correction) for multiple comparisons. Significance level of P < 0.01 or P < 0.05 were indicated by asterisks (^*^) and hashes (#), respectively.

## Supplementary Material

FINAL_all_supplemental_figures_legends_ddaf185
