# Supplementary material for: PTEN variant and genetic backgrounds combine to modify cerebellar neuronal differentiation in autism spectrum disorder
Source: Hum Mol Genet. 2025 Dec 10;35(2):ddaf185. doi: 10.1093/hmg/ddaf185 (PMC13158238; doi:10.1093/hmg/ddaf185)
Supplement: FINAL_all_supplemental_figures_legends_ddaf185 [file final_all_supplemental_figures_legends_ddaf185.pdf]

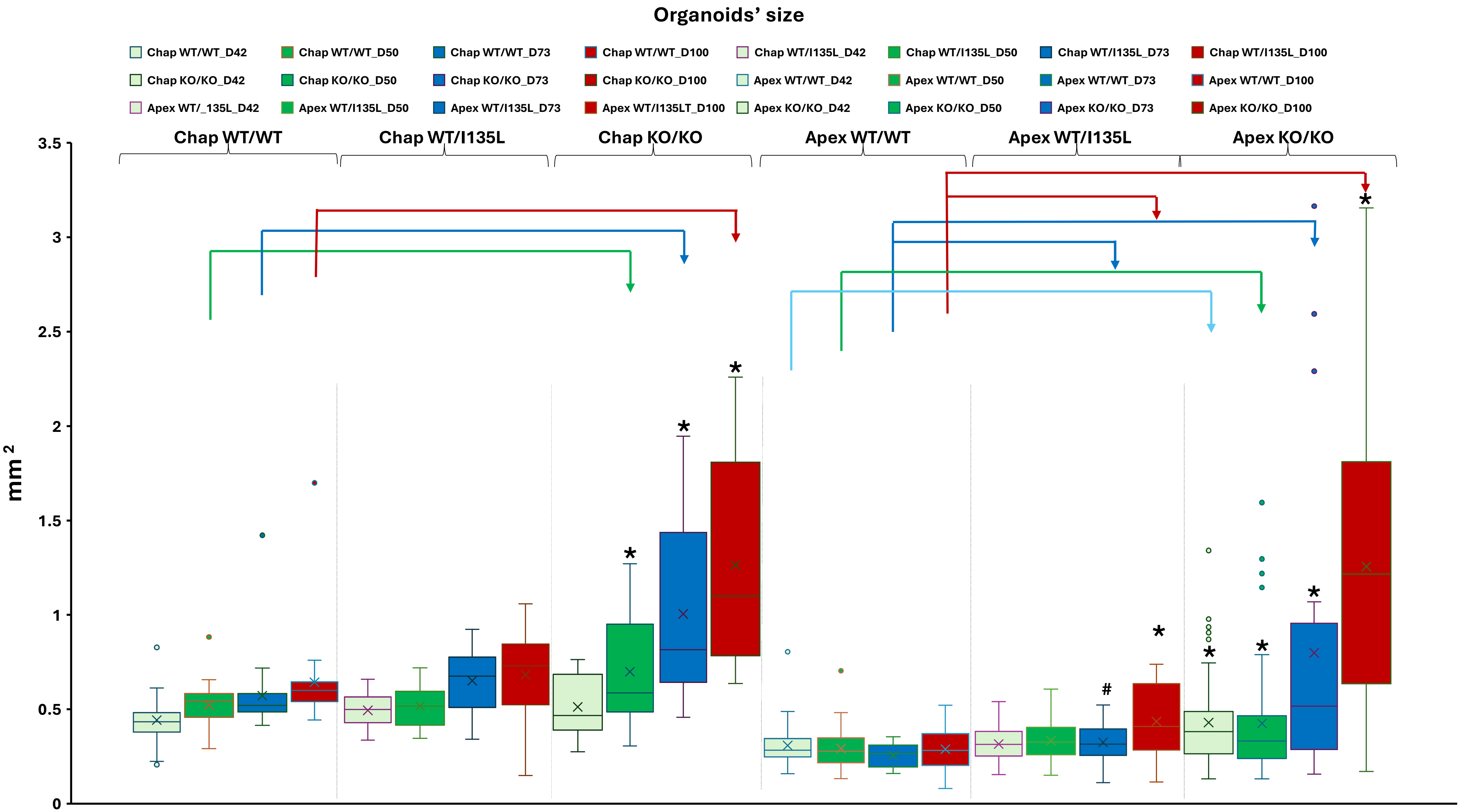

Supplemental figure 1. Organoids sizes were derived from Isogenic PTEN panel iPSCs cerebellar organoids (Chap WT/WT. Chap WT/I135L, Chap KO/KO, Apex WT/WT, Apex WT/I135L, and Apex KO/KO) starting at 9 days up (n=24) to 150 days (n=12). Subsequently, the size of the organoids (mm2) was measured from day 42 (D42, n=24), day 50 (D50, n=24), day 73 (D73, n=24) and day 100 (D100, n=24). Statistical significance indicated by * (P<0.01) and # (P<0.05)

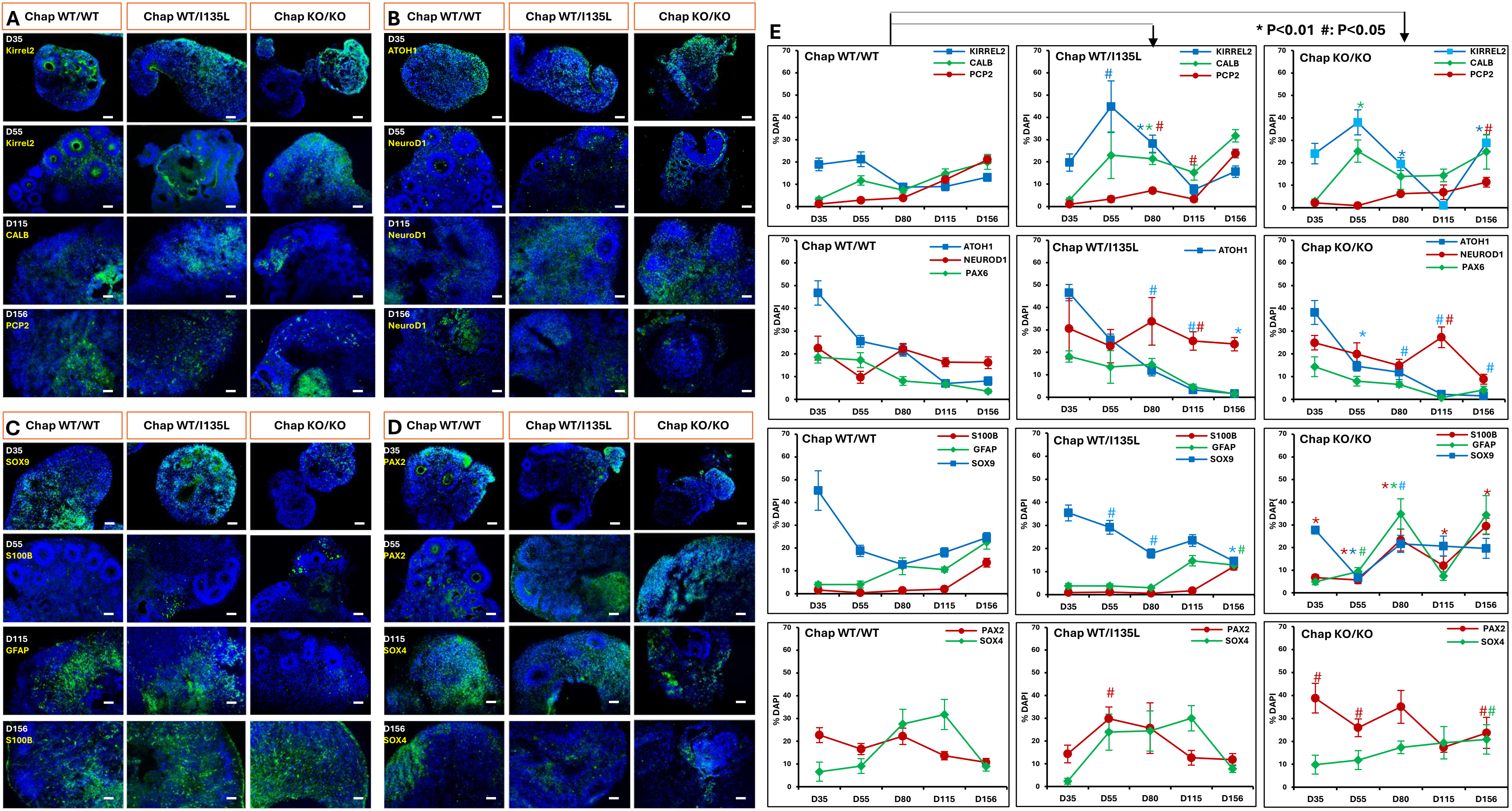

Supplemental figure 2. Isogenic PTEN panel iPSCs in control (Chap) background were differentiated and development cerebellar organoids as experiment 1 (Exp1), including Chap WT/WT, Chap WT/I135L and Chap KO/KO. The whole cerebellar organoids were fixed and sectioned to investigate their spatiotemporal expression patterns at post-differentiation D35, D55, D80, D115, and D156. Immunohistochemistry (IHC) staining was performed and quantified using markers KIRREL2, CALB, PCP2 (A, E); ATOH1, PAX6, NEUROD1 (B, F); S100B, GFAP. SOX9(C, G); and PAX2 and SOX4 (D, H). Scale bar: 20µm. All essential proteins were quantified for expression from 35 days to 156 days in organoids (n>12). (* P<0.01 and # P<0.05, color matched the protein's expression line)

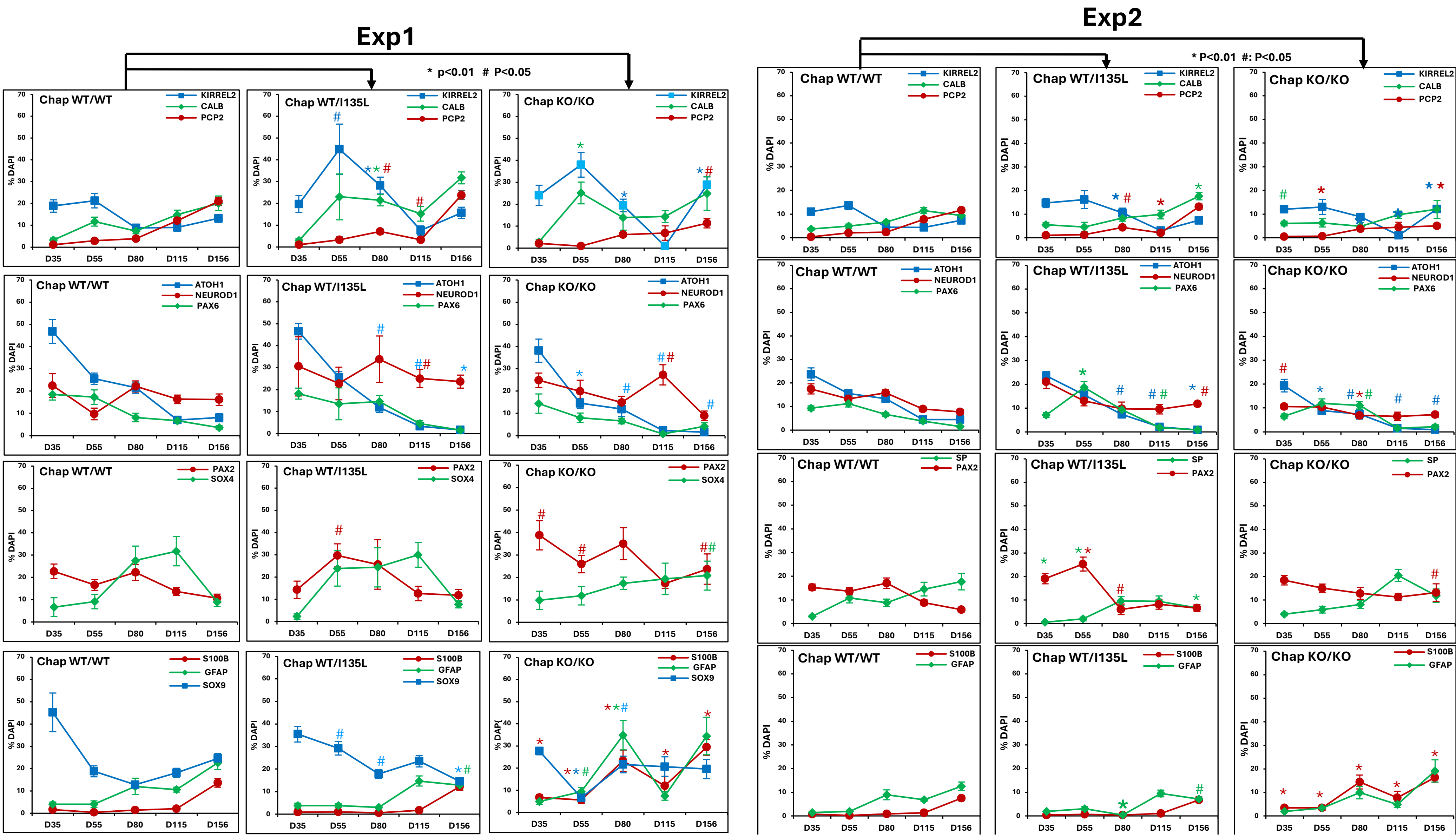

Supplemental figure 3. A comparison was made between two separate datasets from two experiments only involving the Isogenic PTEN panel of iPSCs derived from Chap control background (Fig. 1 and Supplemental Fig. 2). All essential proteins were quantified for expression from 35 days to 156 days in organoids (n>12). (* P<0.01 and # P<0.05, color matched the protein's expression line)

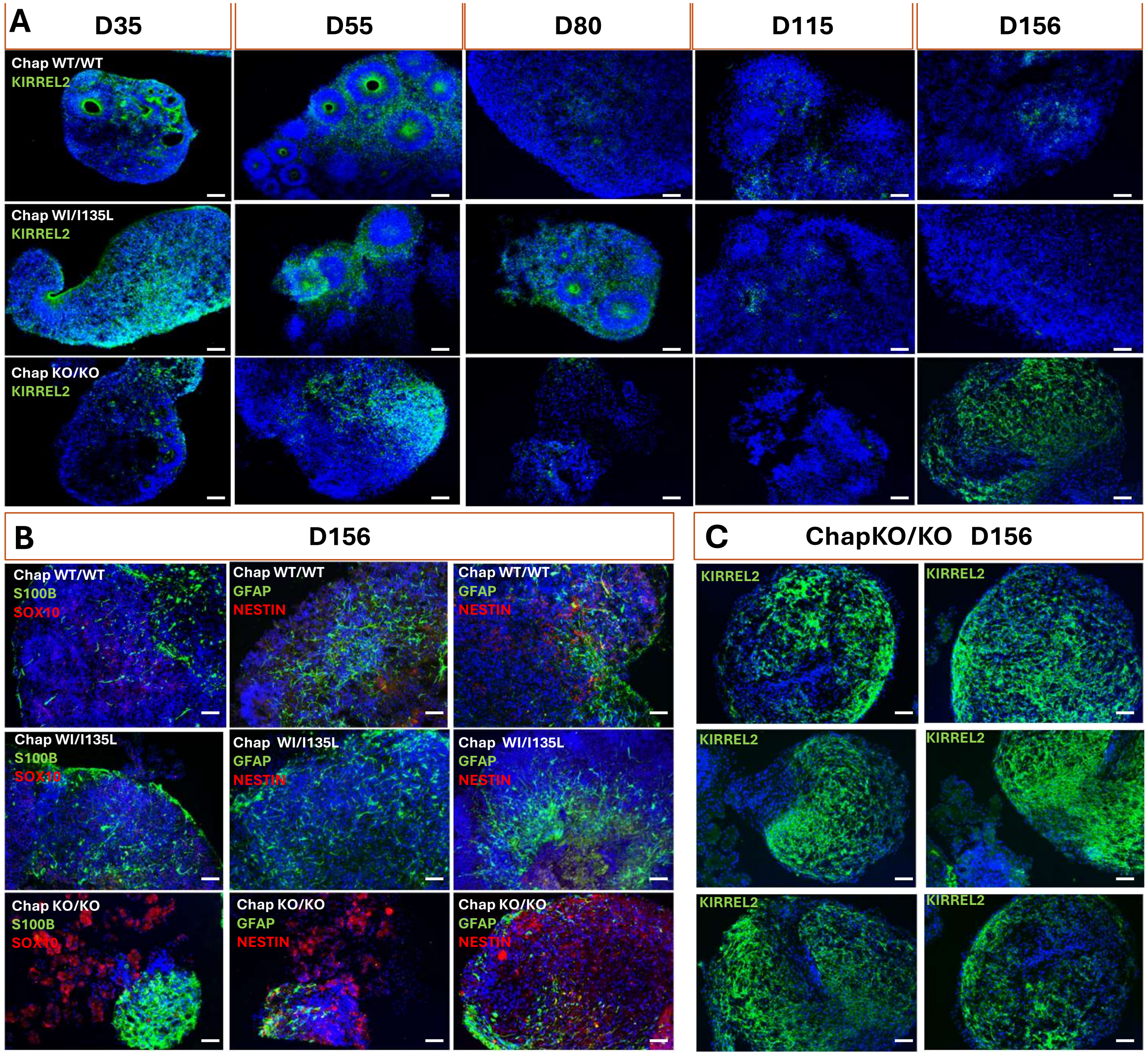

Supplemental figure 4. Neuroepithelial cells reappeared late in Chap KO/KO cerebellar organoids. KIRREL2 was expressed in isogenic Chap WT/WT, Chap WT/I135L and Chap KO/KO organoids from D35 to D80, reduced to D115, and then reappeared only in PTEN KO/KO organoids at D156 (A). At D156, NESTIN and SOX10 were only expressed in Chap KO/KO organoids (B) and more or ganoids expressed KIRREL2 in Chap KO/KO organoids on D156 (C). Scale bar: 20μm.

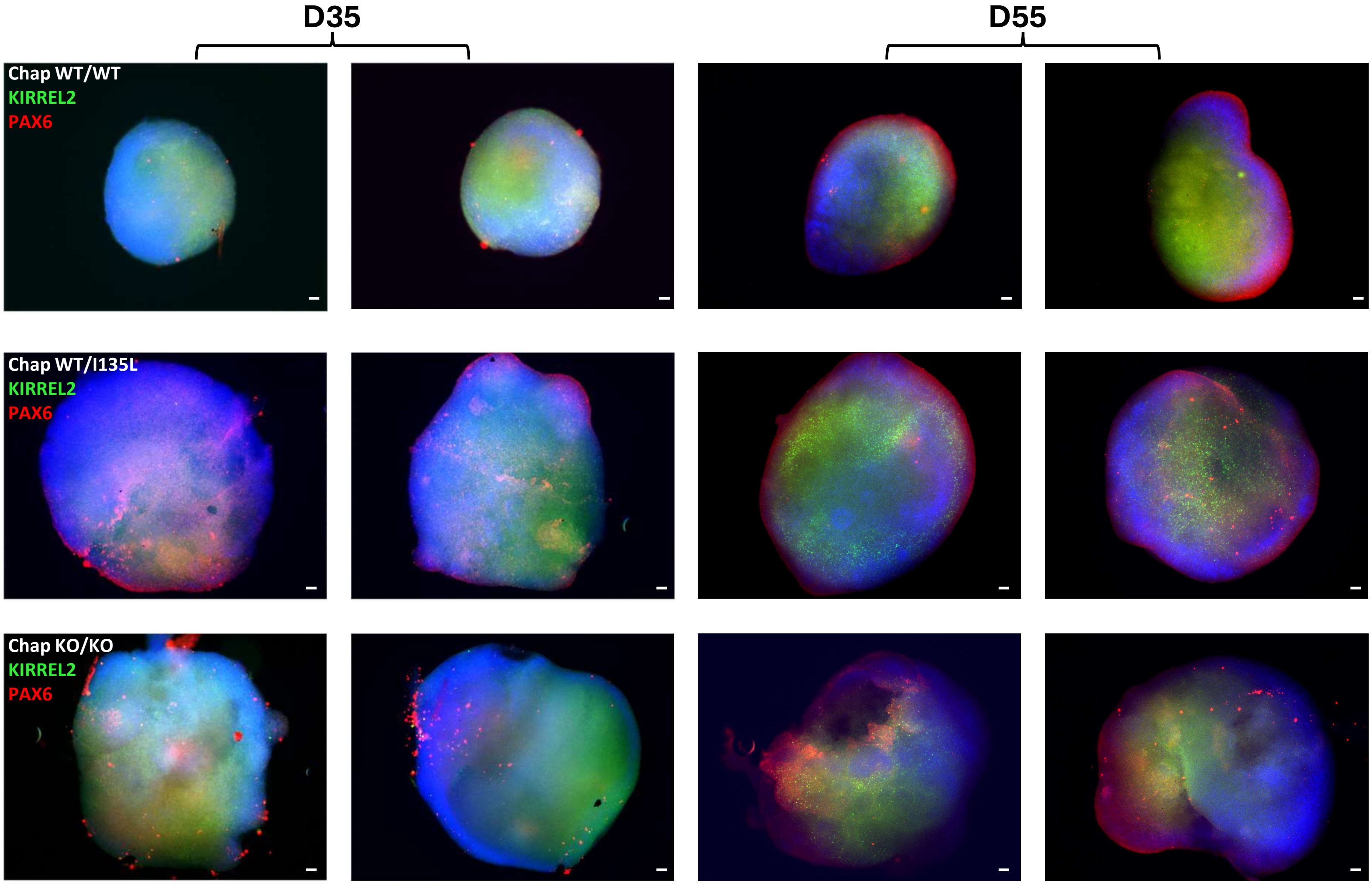

Supplemental figure 5: 3D-stained organoids from isogenic Chap WT/WT, Chap WT/I135L and Chap KO/KO on D35 and D55 with KIRREL2 and PAX6. Only WT/WT organoids displayed polarity between Purkinje cells and granule cells at D55. Scale bar: 20µm

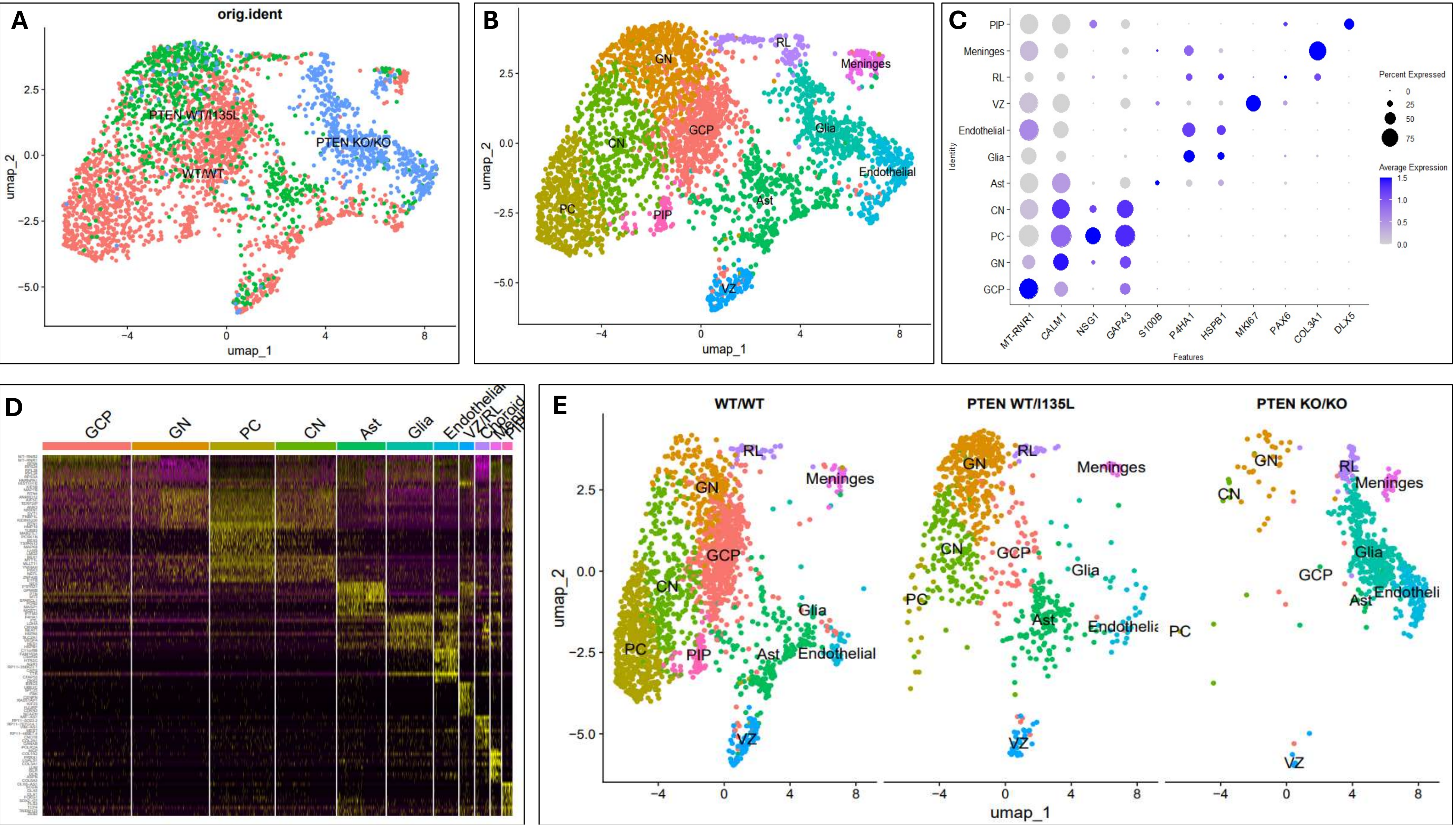

Supplemental figure 6. Single-cell RNA sequencing analysis was performed on the difference between cerebellar organoids derived from WT/WT (Chap WT/WT), PTEN WT/I135L (Chap WT/I135L), and PTEN KO/KO (Chap KO/KO) on 80 days. A: UMAP visualization was utilized to represent cells from the three types of cerebellar organoids, with each individual dataset color-coded WT/WT (n=2081), PTEN WT/I135L (n=836), and PTEN KO/KO (n=618). B: Visualization of the same UMAP plot as in A, but color coded for the 11 classes of cell types found. C: DotPlot depicting gene expressions in different cell types. D: A heatmap was constructed to visualize the characteristic expression patterns of differentially expressed genes per cell population among the 11 identified cell types, filtered by log fold-change values. E: UMAP plots of each of the individual libraries with visualization of the same 11 classes of cell types found in C.

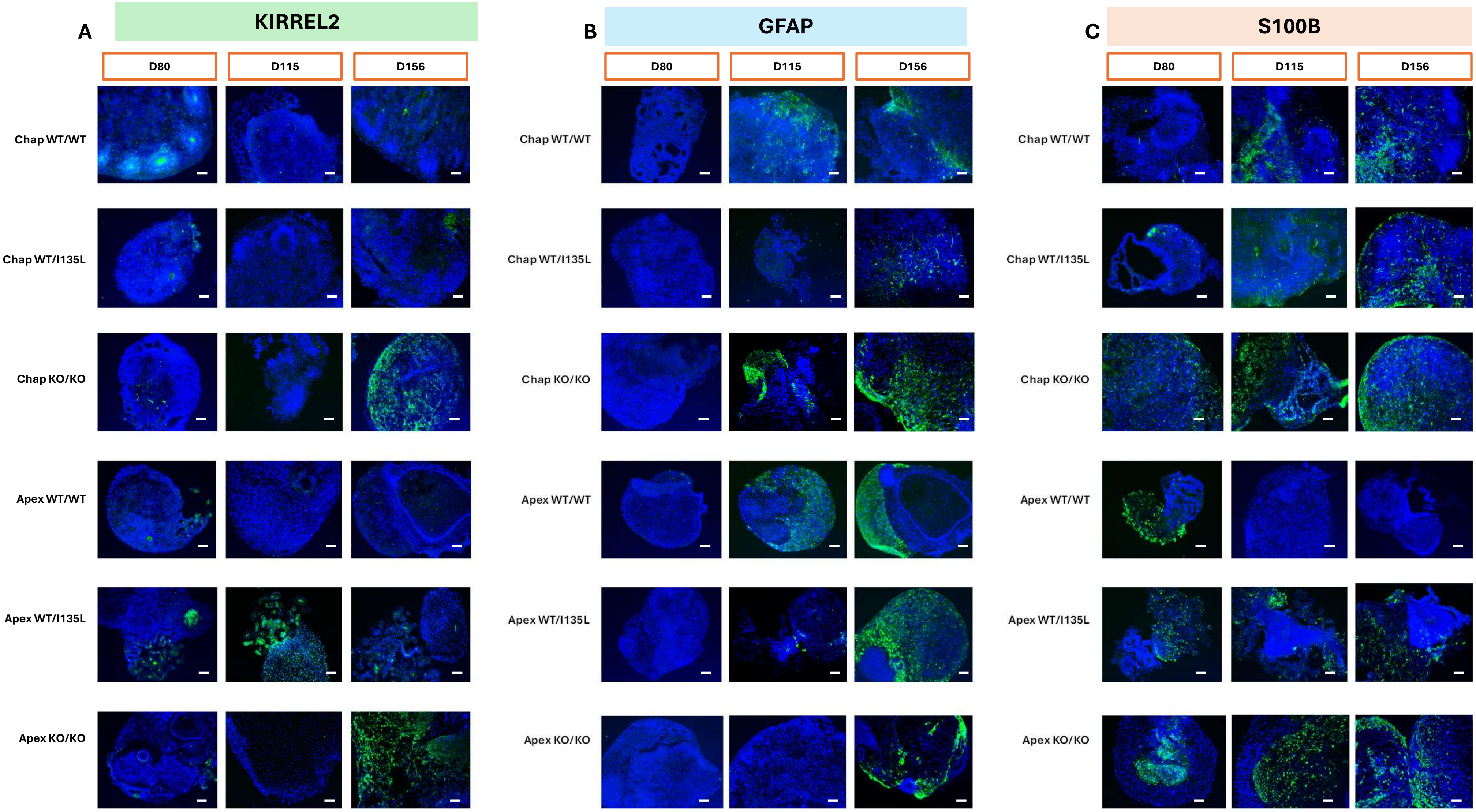

Supplemental figure 7. Isogenic PTEN panel cerebellar organoids in control (Chap) and ASD (Apex) genetic background were fixed at post-differentiation D80, D115, and D156 and sectioned to investigate their spatiotemporal expression patterns of KIRREL2 (A), GFAP (B), and S100B (C).

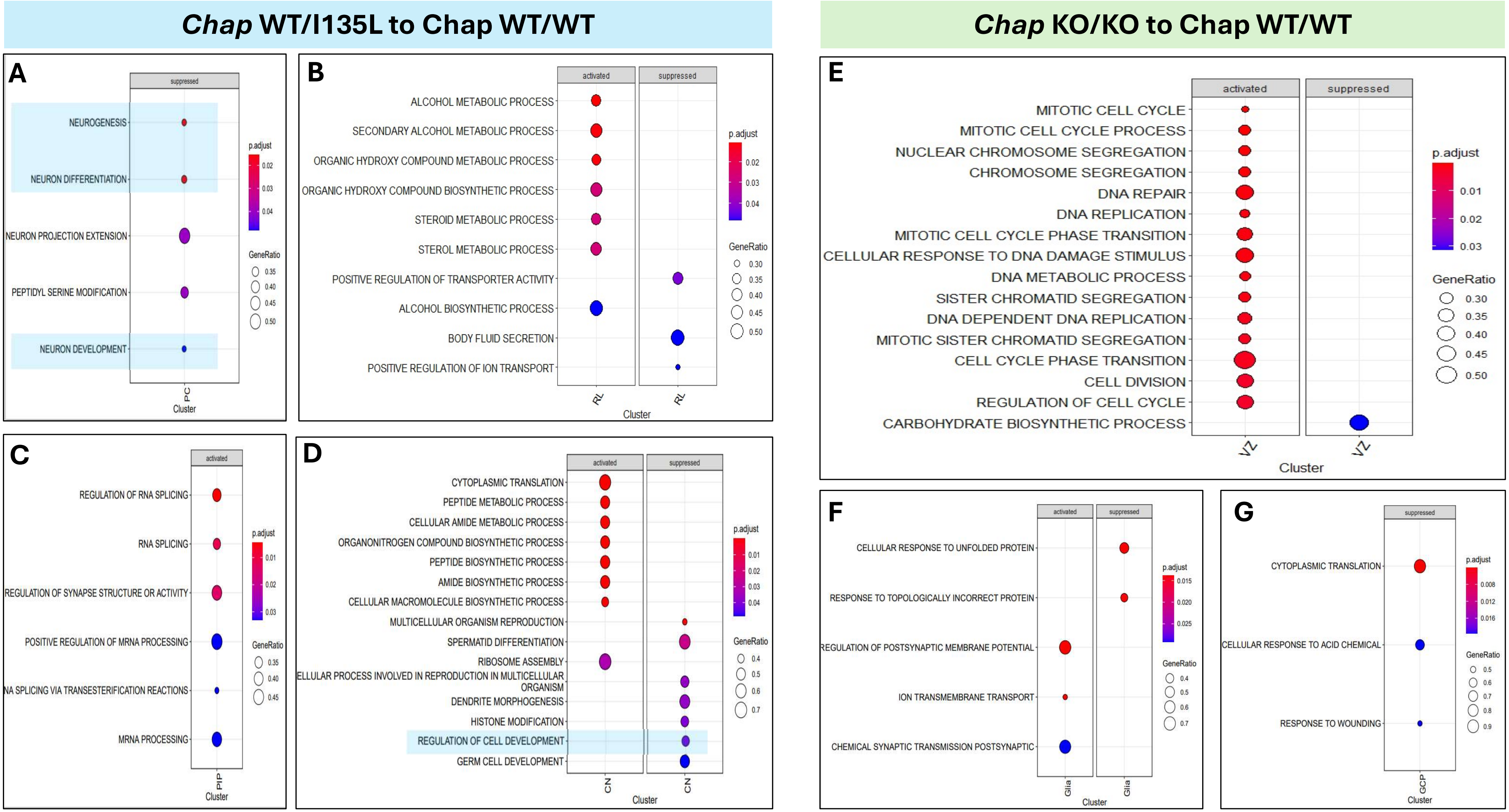

Supplemental figure 8. GSEA for the effect of cells in the control (Chap) background in D80 cerebellar organoids between Chap WT/I135L with Chap WT/WT, PC (A), RL (B), PIP (C), and CN (D); between Chap KO/KO with Chap WT/WT, VZ (E), Glial (F), and GCP (G).

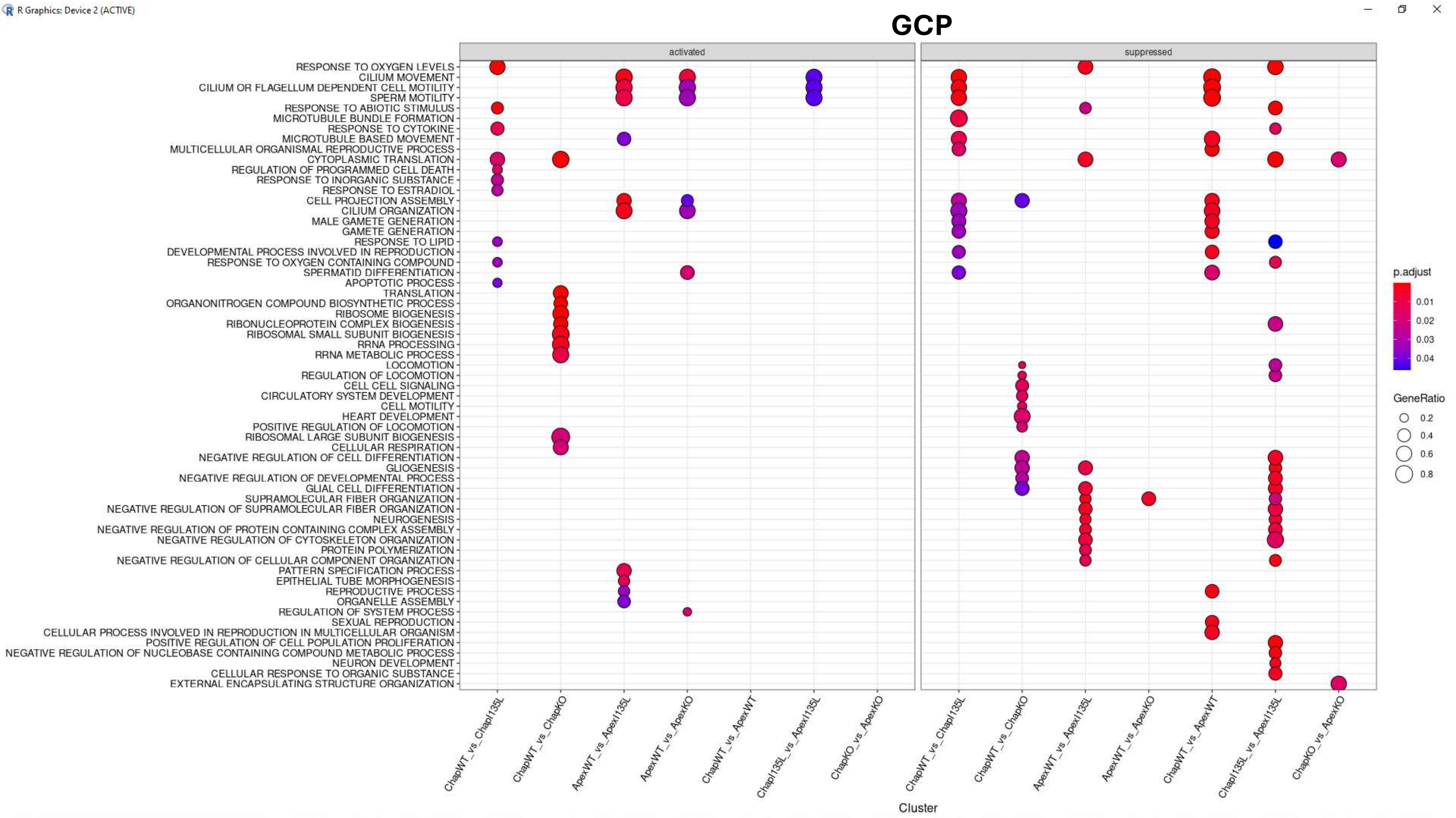

Supplemental figure 9. GSEA for the effect of GCP in the control (Chap) background, ASD (Apex) genetic background and between same mutation from Chap and Apex Isogenic PTEN panel D55 cerebellar organoids, using compare cluster function to visualize the top 10 Gene Ontology (GO) term enrichments.

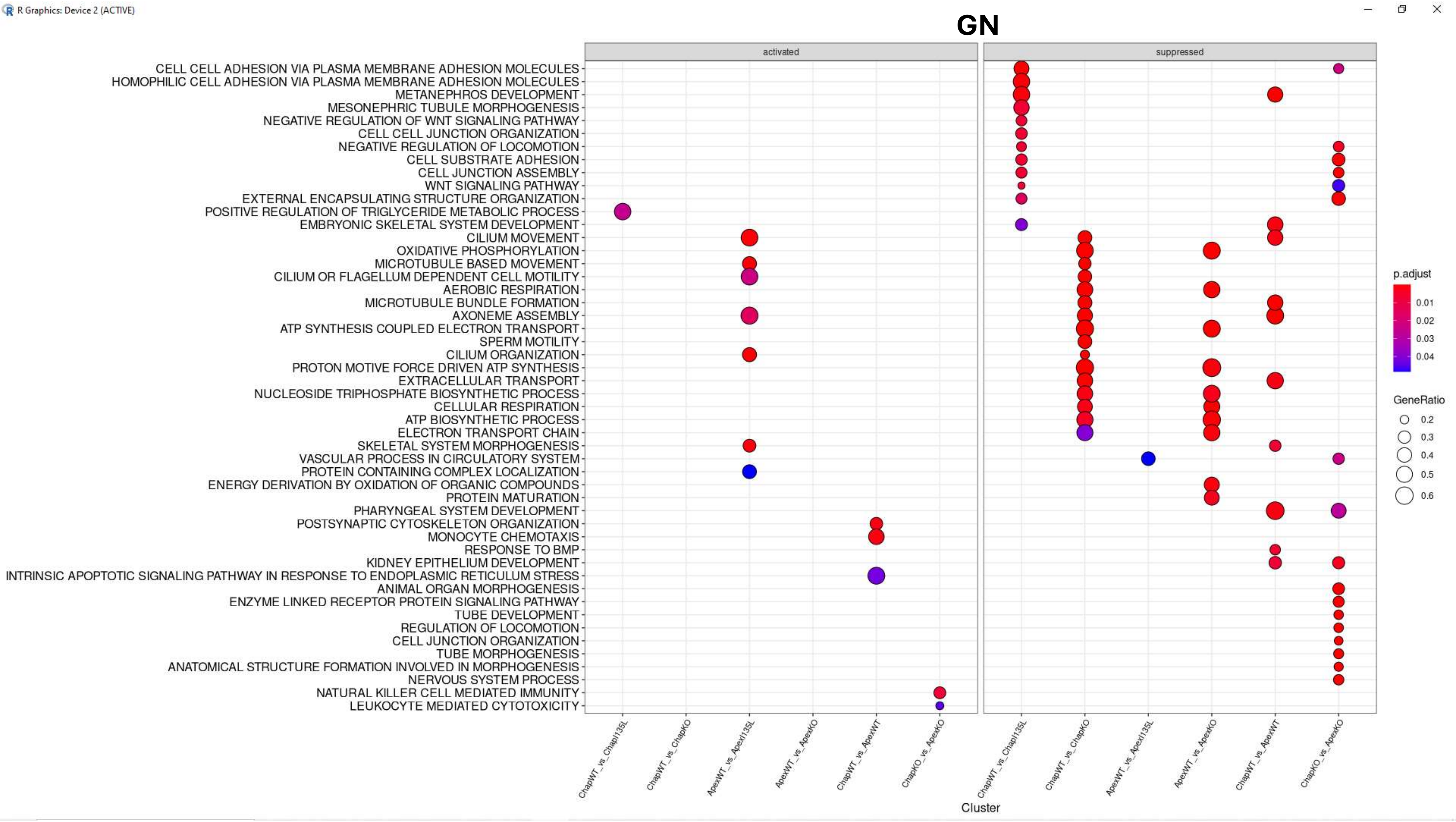

Supplemental figure 10. GSEA for the effect of GN in the control (Chap) background, ASD (Apex) genetic background and between same mutation from Chap and Apex Isogenic PTEN panel D55 cerebellar organoids, using compare cluster function to visualize the top 10 Gene Ontology (GO) term enrichments.

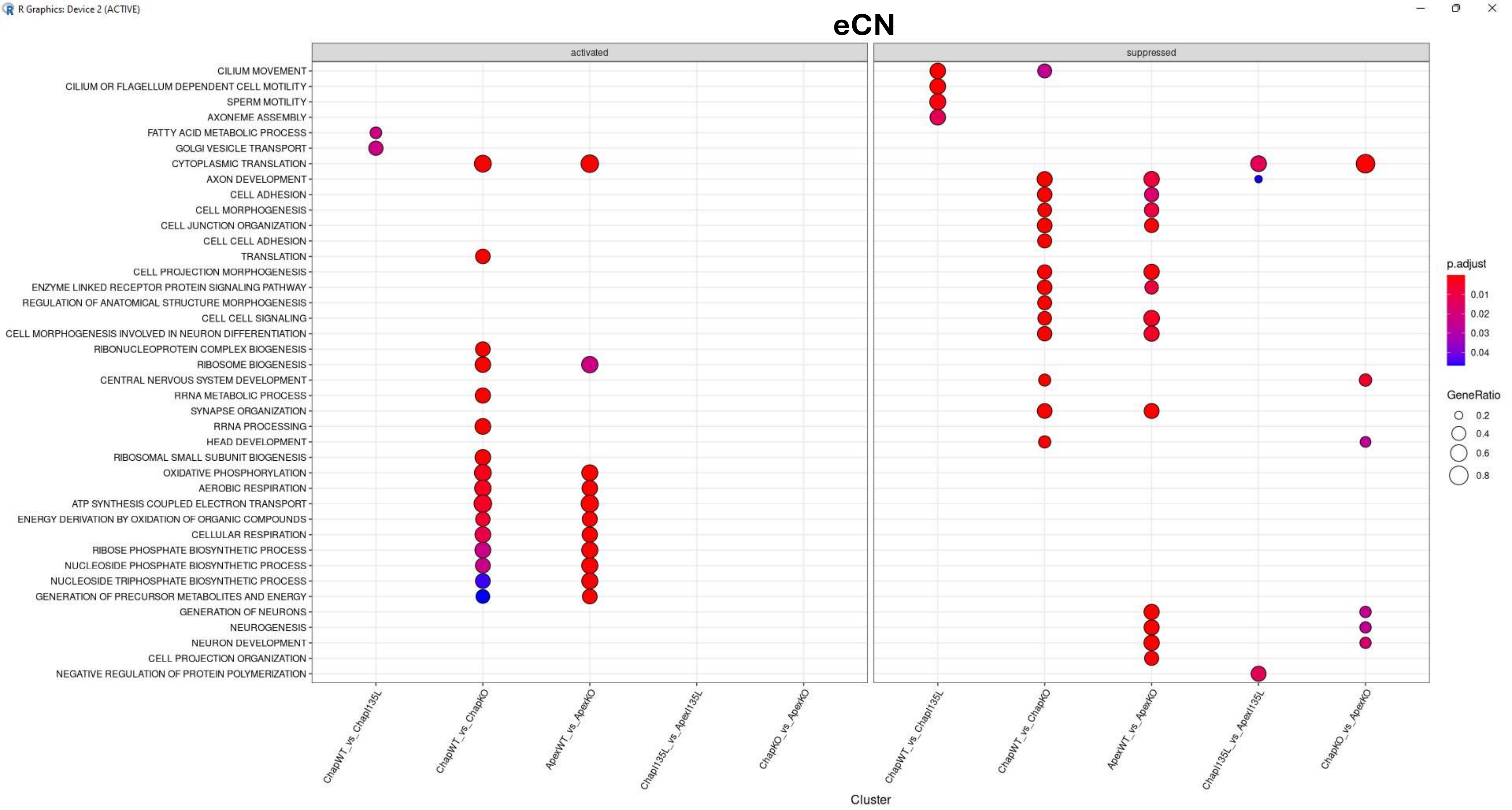

Supplemental figure 11. GSEA for the effect of eCN in the control (Chap) background, ASD (Apex) genetic background and between same mutation from Chap and Apex Isogenic PTEN panel D55 cerebellar organoids, using compare cluster function to visualize the top 10 Gene Ontology (GO) term enrichments.

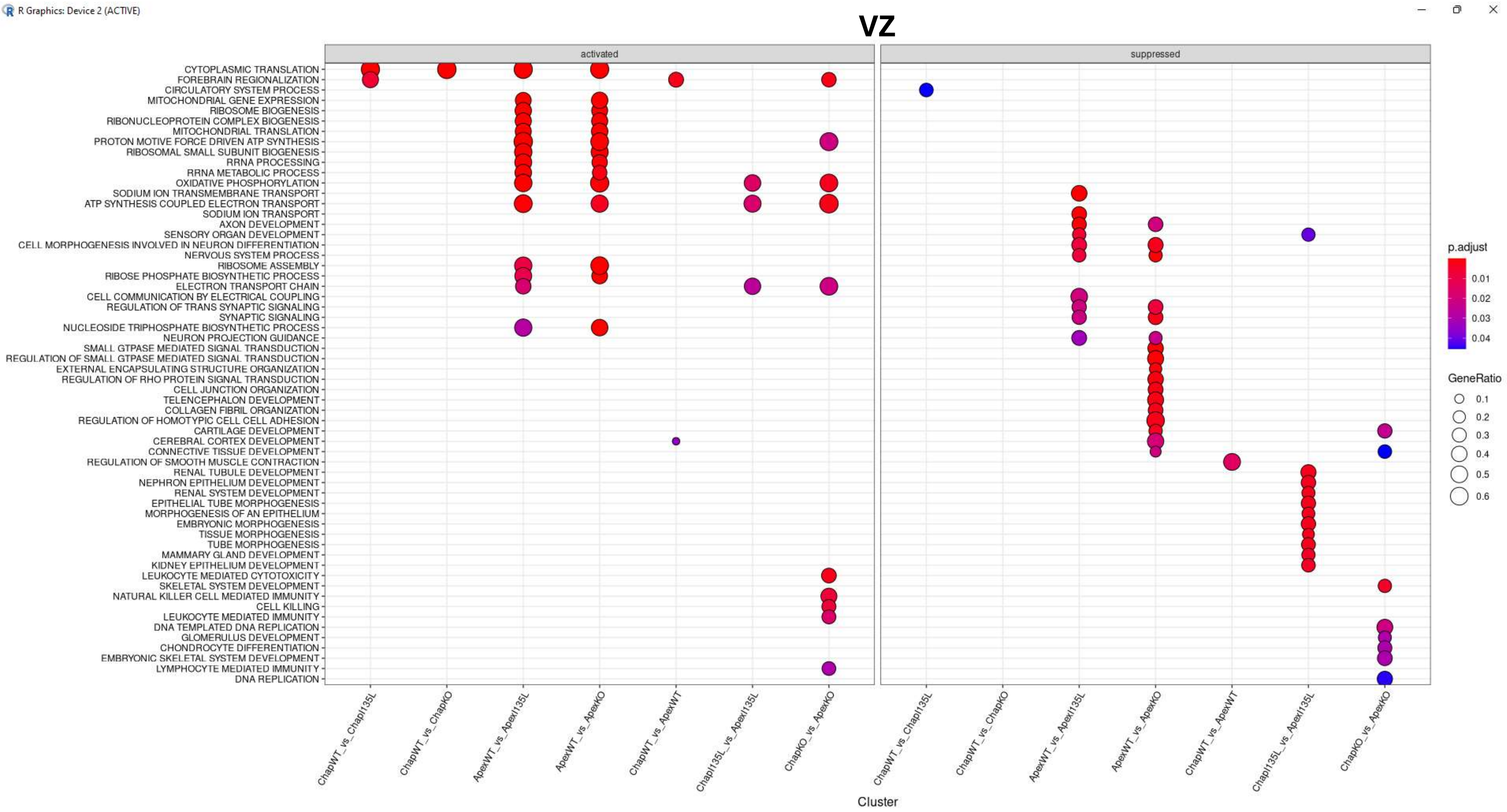

Supplemental figure 12. GSEA for the effect of VZ in the control (Chap) background, ASD (Apex) genetic background and between same mutation from Chap and Apex Isogenic PTEN panel D55 cerebellar organoids, using compare cluster function to visualize the top 10 Gene Ontology (GO) term enrichments.

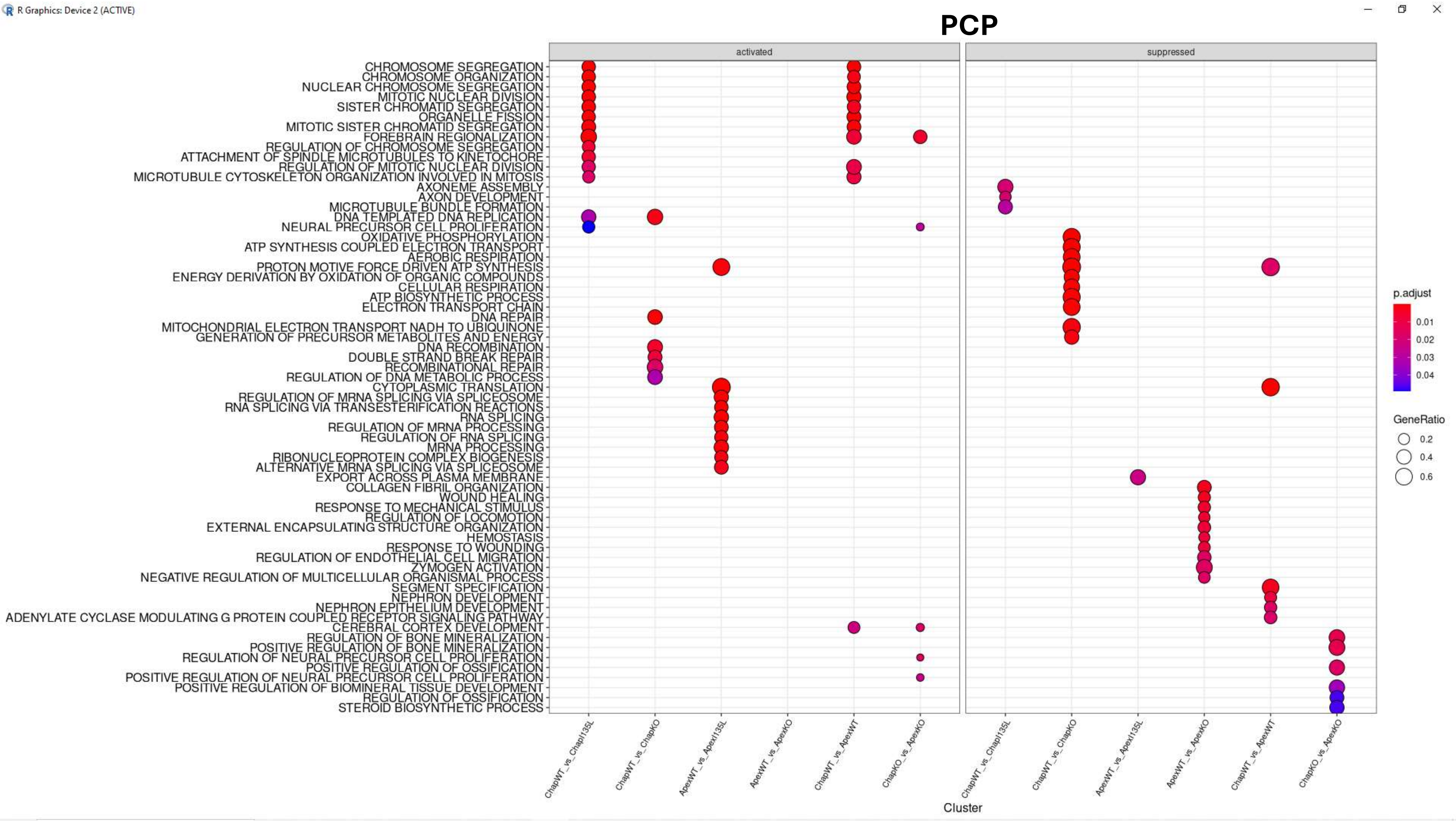

Supplemental figure 13. GSEA for the effect of PCP in the control (Chap) background, ASD (Apex) genetic background and between same mutation from Chap and Apex Isogenic PTEN panel D55 cerebellar organoids, using compare cluster function to visualize the top 10 Gene Ontology (GO) term enrichments.

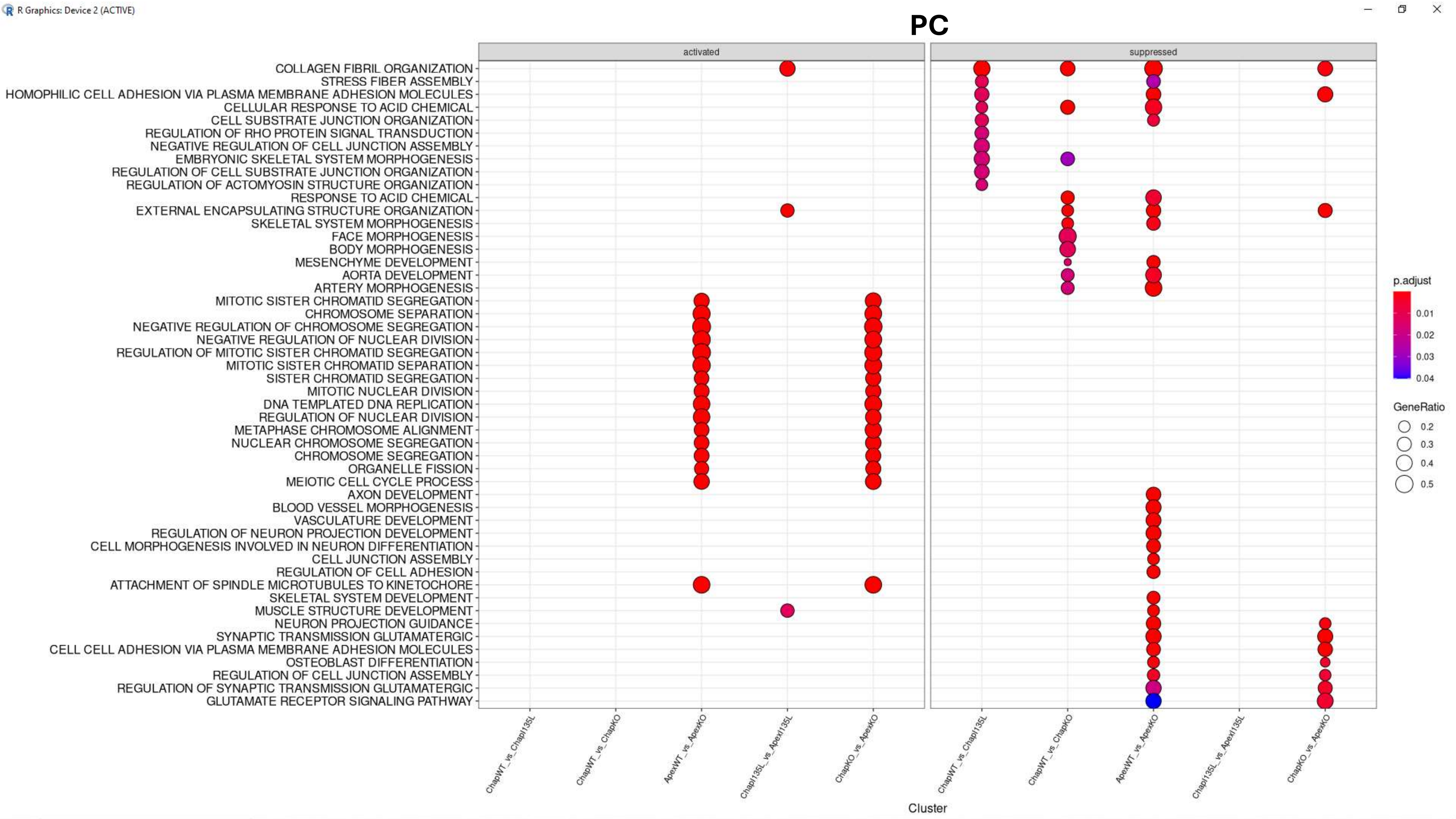

Supplemental figure 14. GSEA for the effect of PC in the control (Chap) background, ASD (Apex) genetic background and between same mutation from Chap and Apex Isogenic PTEN panel D55 cerebellar organoids, using compare cluster function to visualize the top 10 Gene Ontology (GO) term enrichments.

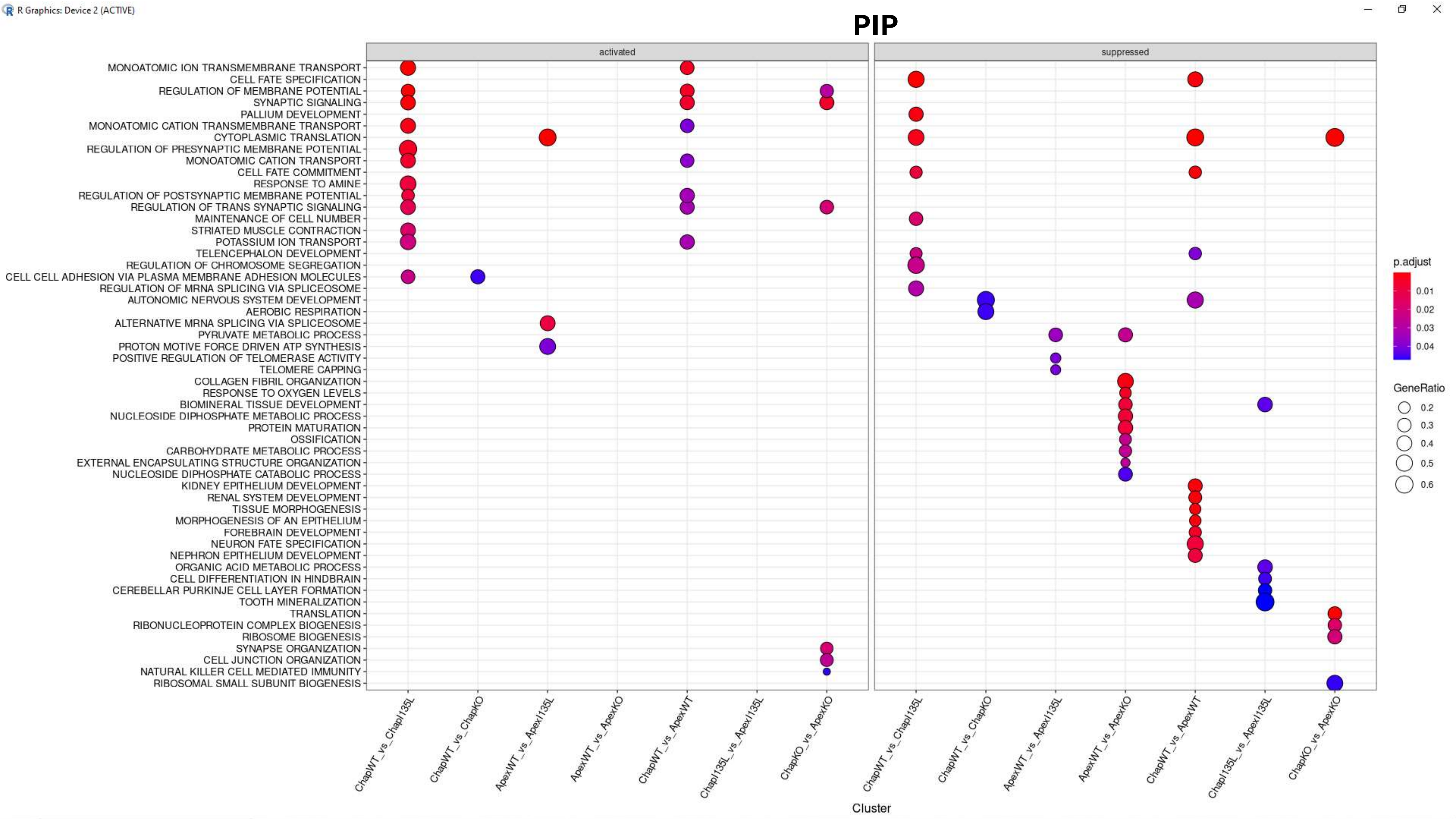

Supplemental figure 15. GSEA for the effect of PIP in the control (Chap) background, ASD (Apex) genetic background and between same mutation from Chap and Apex Isogenic PTEN panel D55 cerebellar organoids, using compare cluster function to visualize the top 10 Gene Ontology (GO) term enrichments.

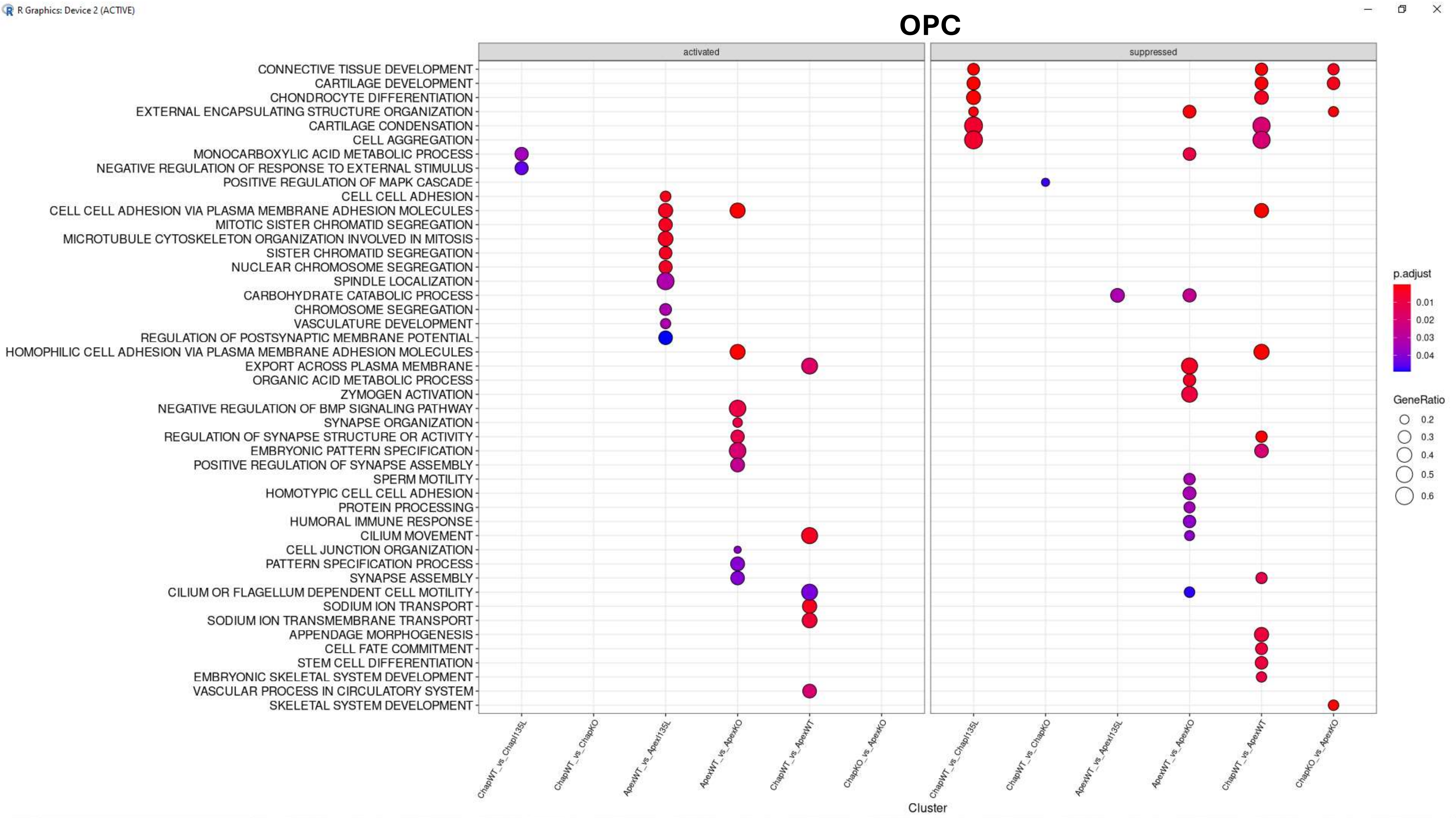

Supplemental figure 16. GSEA for the effect of OPC in the control (Chap) background, ASD (Apex) genetic background and between same mutation from Chap and Apex Isogenic PTEN panel D55 cerebellar organoids, using compare cluster function to visualize the top 10 Gene Ontology (GO) term enrichments.

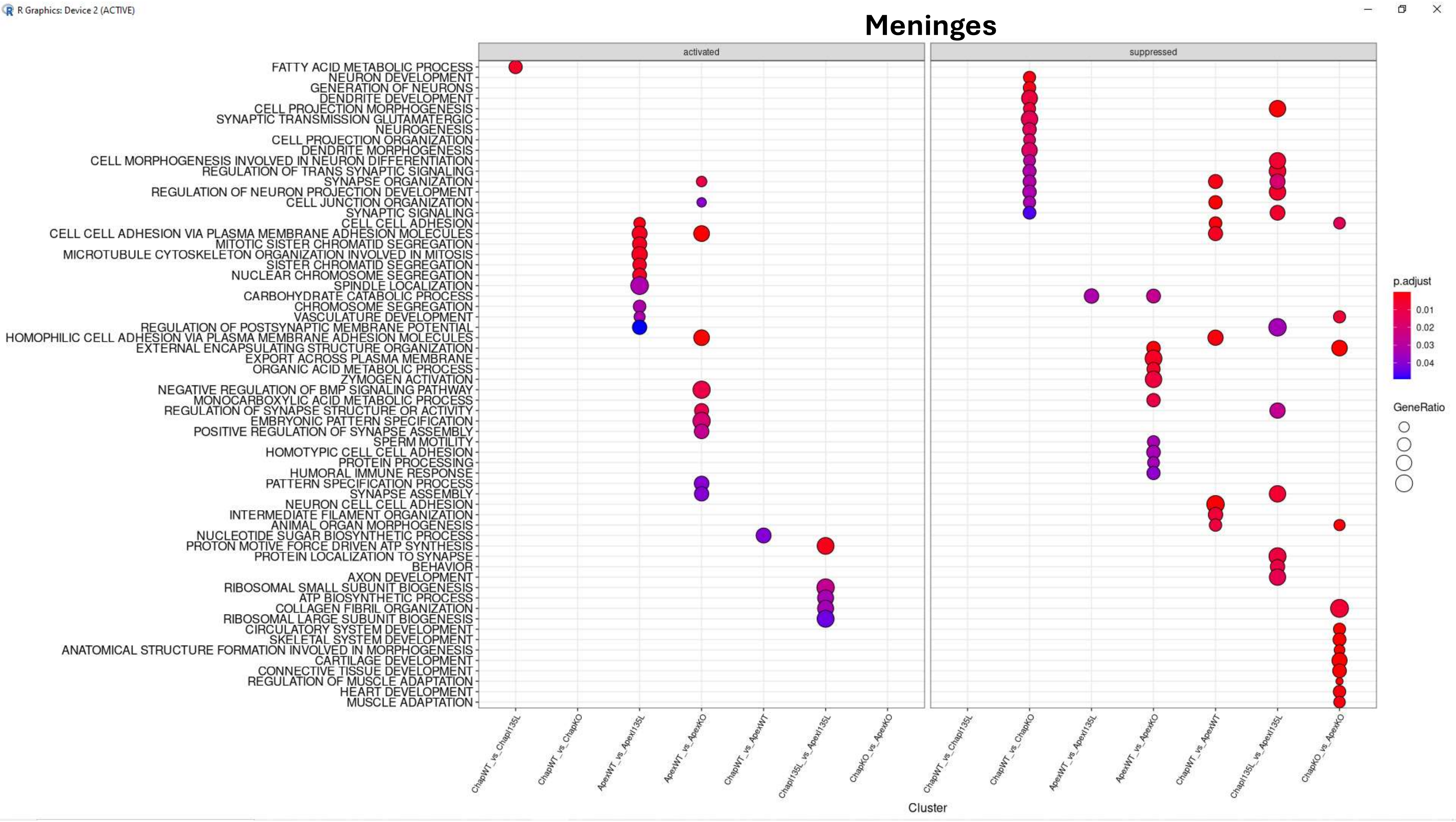

Supplemental figure 17. GSEA for the effect of meninges in the control (Chap) background, ASD (Apex) genetic background and between same mutation from Chap and Apex Isogenic PTEN panel D55 cerebellar organoids, using compare cluster function to visualize the top 10 Gene Ontology (GO) term enrichments.

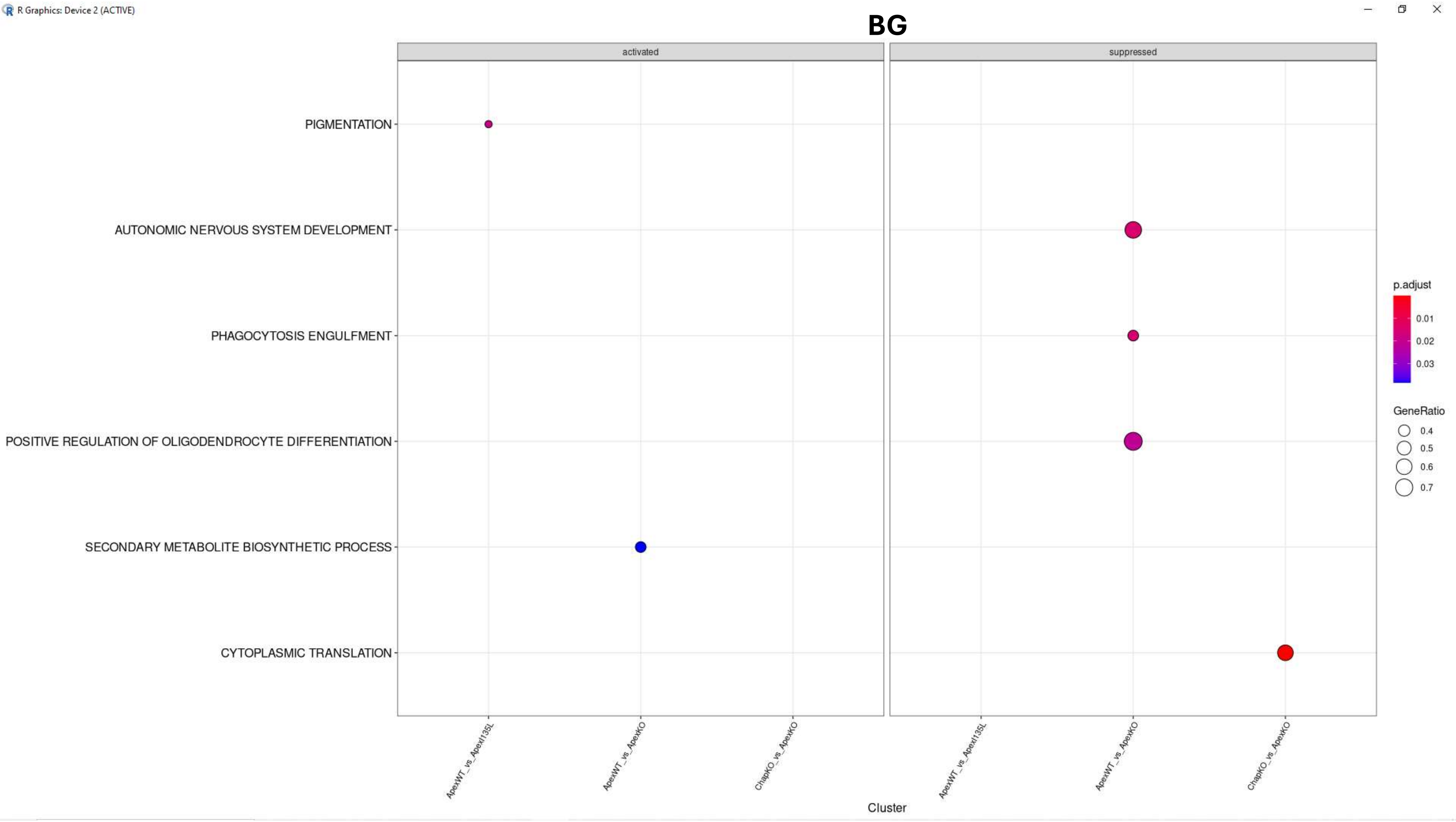

Supplemental figure 18. GSEA for the effect of BG in the control (Chap) background, ASD (Apex) genetic background and between same mutation from Chap and Apex Isogenic PTEN panel D55 cerebellar organoids, using compare cluster function to visualize the top 10 Gene Ontology (GO) term enrichments.
